# Targeted Radium Alpha Therapy in the Era of Nanomedicine: In Vivo Results

**DOI:** 10.3390/ijms25010664

**Published:** 2024-01-04

**Authors:** György Trencsényi, Csaba Csikos, Zita Képes

**Affiliations:** 1 Division of Nuclear Medicine and Translational Imaging, Department of Medical Imaging, Faculty of Medicine, University of Debrecen, Nagyerdei St. 98, H-4032 Debrecen, Hungary; trencsenyi.gyorgy@med.unideb.hu (G.T.); csikosc@icloud.com (C.C.); 2 Gyula Petrányi Doctoral School of Clinical Immunology and Allergology, Faculty of Medicine, University of Debrecen, Nagyerdei St. 98, H-4032 Debrecen, Hungary

**Keywords:** nanoparticles, preclinical, Radium-223/224 (^223/224^Ra), targeted alpha-particle therapy, xenotransplants

## Abstract

Targeted alpha-particle therapy using radionuclides with alpha emission is a rapidly developing area in modern cancer treatment. To selectively deliver alpha-emitting isotopes to tumors, targeting vectors, including monoclonal antibodies, peptides, small molecule inhibitors, or other biomolecules, are attached to them, which ensures specific binding to tumor-related antigens and cell surface receptors. Although earlier studies have already demonstrated the anti-tumor potential of alpha-emitting radium (Ra) isotopes—Radium-223 and Radium-224 (^223/224^Ra)—in the treatment of skeletal metastases, their inability to complex with target-specific moieties hindered application beyond bone targeting. To exploit the therapeutic gains of Ra across a wider spectrum of cancers, nanoparticles have recently been embraced as carriers to ensure the linkage of ^223/224^Ra to target-affine vectors. Exemplified by prior findings, Ra was successfully bound to several nano/microparticles, including lanthanum phosphate, nanozeolites, barium sulfate, hydroxyapatite, calcium carbonate, gypsum, celestine, or liposomes. Despite the lengthened tumor retention and the related improvement in the radiotherapeutic effect of ^223/224^Ra coupled to nanoparticles, the in vivo assessment of the radiolabeled nanoprobes is a prerequisite prior to clinical usage. For this purpose, experimental xenotransplant models of different cancers provide a well-suited scenario. Herein, we summarize the latest achievements with ^223/224^Ra-doped nanoparticles and related advances in targeted alpha radiotherapy.

## 1. Introduction

### 1.1. Targeted Alpha Radiotherapy

Because the shortcomings of conventional radiotherapy [1,2,3] limit the attractiveness of standard oncological treatment approaches, attempts have been made to optimize the delivery of radiation therapy to cancer cells. Recently, targeted radionuclide therapy (TRT) using alpha emitters has gained remarkable attention for cancer treatment [4].

Due to their favorable physicochemical properties, alpha emitter radionuclides are capable of exerting cellular cytotoxicity on tumor cells, thus causing minimal exposure to the healthy tissues nearby [5]. Complex DNA disruptions [6,7] induced by the high linear energy (LET: 50–230 keV/μm) of alpha rays are responsible for a significant tumor cell-killing effect, while the short path of the particles (50–100 μm) ensures the protection of the surrounding intact organs [5,8]. Beyond DNA damage, alpha particles cause direct alterations to other cellular compartments, such as the mitochondria, lysosomes, and the cell membranes, which, along with bystander and immunological effects, have a central role in the achievement of alpha-mediated anti-tumor effect [9,10,11]. The short penetration within tissue favors the eradication of micrometastatic and residual lesions as well as single malignant cells [12]. Attached to target-specific carriers (for example, antibodies, peptides, or small molecules), alpha emitters are able to selectively irradiate all neoplastic lesions, including the primary tumors as well as their metastases irrespective of their location [13,14,15]. In addition, the radiotoxicity of high-LET alpha particles is independent of oxygenation; therefore, radioisotopes with alpha emission are considered suitable for destroying tumor masses even under hypoxic conditions [16,17]. Overall, these characteristics validate that alpha particles have greater biological effectiveness compared to other radiations in therapeutic settings. 

**Figure 1:**
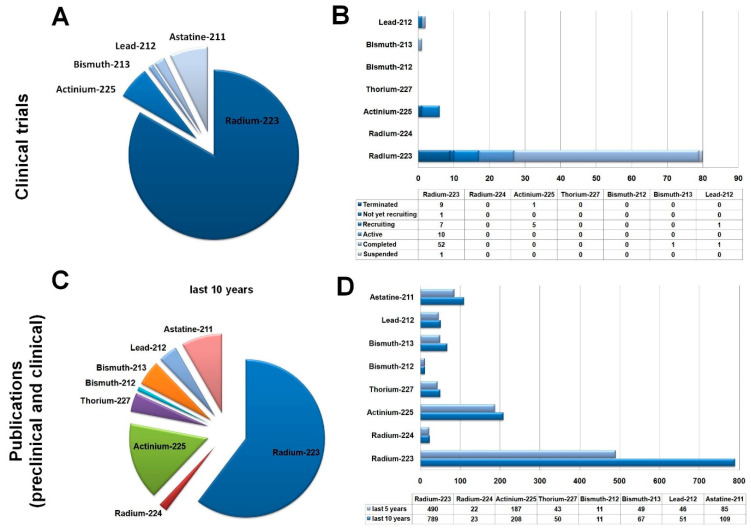
Statistical analysis of the clinical trials (**A**,**B**) and the number of preclinical and clinical publications (**C**,**D**) on ^223^Ra, ^224^Ra, ^225^Ac, ^227Th^, ^212^Bi, ^213^Bi, ^212^Pb, and ^211^At isotopes. (**A**) The percentage distribution (%) of different alpha-emitting radionuclides in clinical studies—available to date—investigating the anti-tumor efficacy of alpha radiation: ^223^Ra (84%), ^224^Ra (0%), ^225^Ac (6%), ^227Th^ (0%), ^212^Bi (0%), ^213^Bi (1%), ^212^Pb (2%), and ^211^At (7%). Data obtained from clinicaltrials.gov. (**B**) Detailed status analysis of the clinical trials using the investigated alpha-emitting radionuclides. Data obtained from clinicaltrials.gov. (**C**) Distribution of the published preclinical and clinical scientific papers in the field of alpha-emitting radionuclide therapies in the last 10 years. Data obtained from pubmed.ncbi.nlm.nih.gov. (**D**) The number of preclinical and clinical publications on targeted alpha radionuclide therapies from the last 5 and 10 years. Data obtained from pubmed.ncbi.nlm.nih.gov. ^223^. Ra: Radium-223, ^224^Ra: Radium-224, ^225^Ac: Actinium-225, ^227Th^: Thorium-227, ^212^Bi: Bismuth-212, ^213^Bi: Bismuth-213, ^212^Pb: Lead-212, ^211^At: Astatine-211.

Experiments with preclinical model systems represent the cornerstone to gaining deeper insight into the mechanism of action of TRT and the assessment of long-term treatment response. Findings derived from such investigations are not only transferable to in-human patient care but may also lay the groundwork for the identification of novel cellular targets for radiation purposes. The therapeutic value/suitability of targeted alpha treatment was evidenced by a vast array of prior in vitro and in vivo studies [5,18,19,20,21,22]. Within the alpha-emitting radionuclide domain, a broad set of isotopes are available for TRT usage, including, for instance, Radium-223 (^223^Ra), Radium-224 (^224^Ra), Actinium-225 (^225^Ac), Thorium-227 (^227Th^), Bismuth-212 (^212^Bi), Bismuth-213 (^213^Bi), Lead-212 (212Pb), and Astatine-211 (^211^At) [16]. Figure 1 provides statistics from the current clinical trials and the number of preclinical and clinical publications on ^223^Ra, ^224^Ra, ^225^Ac, ^227Th^, ^212^Bi, ^213^Bi, ^212^Pb, and ^211^At isotopes.

### 1.2. Radium-223/Radium-224 (^223/224^Ra)

**Figure 2:**
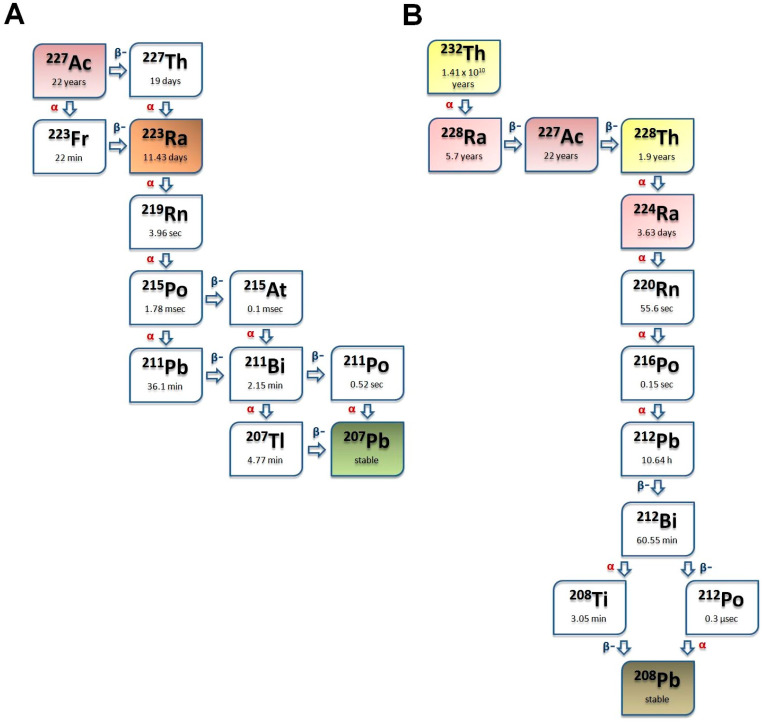
Decay scheme of Radium-223 and Radium-224. Panel (**A**) presents the decay scheme of Radium-223. Panel (**B**) demonstrates the decay scheme of radium-224.

With regard to the positive physicochemical features of alpha-emitting Radium (Ra) isotopes, tremendous research has been undertaken to explore their capability in targeted therapeutic settings [23,24,25]. Due to its high skeletal affinity, Ra is widely used in the treatment of bone metastases [26,27]. ^223^Ra, for example, has been successfully applied for alleviating bone pain due to metastases in breast or prostate cancer patients [28]. Figure 2A,B demonstrate the decay characteristics of two Ra isotopes: ^223^Ra and ^224^Ra. 

Out of the radionuclides of Ra, ^224^Ra, seems to be a potential candidate for therapeutic purposes as an alpha emitter [23,29,30,31]. Previous clinical research revealed pain reduction induced by ^224^Ra treatment in patients with ankylosing spondylitis [32,33]. So far, however, there is little research related to the application of ^224^Ra in oncological settings [30,34]. It could be acquired from Thorium-228 (^228^Th) generators [35]. Compared to other short-lived alpha radioisotopes, such as ^213^Bi (T_1/2_ = 46 min), ^211^At (T_1/2_ = 7.2 h), and ^212^Pb (T_1/2_ = 10.6 h), the longer half-life of ^224^Ra (3.631 (2) days) enables easy transport to distant medical facilities; hence, the establishment of an on-site radiochemical laboratory is not a prerequisite for ^224^Ra production [36,37]. Being an alpha emitter, ^224^Ra possesses high LET values and a short tissue range, which are necessary for the induction of an adequate radiotoxic effect. ^224^Ra is often called a nanogenerator, as it decays to Lead-208 (^208^Pb) via six daughter nuclides involving Radon-220 (^220^Rn), Polonium-216 (^216^Po), ^212^Pb, ^212^Bi, Polonium-212 (^212^Po), and Thallium-208 (^208^Tl) [38]. Decaying through the emission of four alpha and two beta particles with total alpha energies of approximately 26 MeV [31,39], ^224^Ra allows for the transport of sufficient doses to the target areas with low amounts of administered radioactivity. Moreover, the alpha-emitting decay progenies of ^224^Ra (Thoron/220-Radon/^220^Rn; ^216^Po; ^212^Pb/^212^Bi; ^212^Po) could also have meaningful therapeutic implications, which might contribute to the enhancement of the overall anti-tumor effect [40,41]. In addition, the limited accessibility of ^211^At, and the high amount of therapeutically required relevant doses of ^213^Bi, also speak in favor of the application of ^224^Ra [42,43]. The decay scheme of ^224^Ra is displayed in Figure 2B.

Beyond ^224^Ra, ^223^Ra is also emerging as a valuable radionuclide for targeted tumor treatment [44,45]. Acting like a calcium mimetic, ^223^Ra shows increased complexation with skeletal calcium hydroxyapatite crystals in areas of active bone formation [46,47]. ^223^Ra could be eluted from Actinium-227/Thorium-227 (^227^Ac/^227^Th) generator system or it can be obtained from uranium mill tailings [48]. Its multiple-step decay (4 α, 2 β and 5 γ rays) leads to the following emitted energy distributions: 26.87 MeV, 0.94 MeV, and 1.996 MeV for α, β and γ particles, respectively [49]. Figure 2A presents the decay series of ^223^Ra. The high LET value of ^223^Ra ensures that it exerts its cytotoxicity through the induction of double-strand DNA breaks [50]. Similarly to ^224^Ra, due to the high LET value (approximately 28 MeV) and the short path of ^223^Ra alpha rays (60–100 µm), relatively small radioactive doses are sufficient for the achievement of substantial therapeutic effect [24]. This is of paramount importance for both personnel and patients regarding the reduction in their exposure to radiation. Similarly to daughter ^224^Ra, the alpha rays of ^223^Ra decay products could also be therapeutically exploited (^219^Rn, ^215^Po, ^211^Bi), thereby leading to improved anti-tumor-killing effect. Its broad accessibility compared to other alpha emitters such as ^225^Ac, ^211^At, or ^213^Bi, along with the economically viable synthesis from ^227^Ac generators, allow the performance of a wide array of preclinical experiments as well as clinical trials possible [51,52]. The long half-life (T_1/2_ = 11.43 days) of ^223^Ra and the relatively large amounts obtained upon a single elution ensure easy shipment to distant locations, which further contributes to the commercial distribution and the centralized production of both the isotope and its radiopharmaceuticals [53,54,55]. In a practical sense, the longevity of ^223^Ra also gives enough room for synthesis, radiolabeling, handling, and quality control [53,56]; moreover, it could be seamlessly customized to the half-time of larger molecules including peptides or nanoconjugates. 

In spite of the promising features of ^223/224^Ra, tackling with the difficulty to deliver these radioisotopes into the intended neoplastic sites is an issue of major concern. Hence, to exploit the favorable properties of Ra in cancer treatment, exhaustive research has been centered around the investigation of suitable carriers including bifunctional chelating agents and other delivery systems that can bind Ra to target-specific molecules [57,58,59,60,61,62]. These transport molecules would also enable the extension of the use of Ra isotopes beyond bone-seeking purposes. To select the best Ra chelates, a vast array of studies assessing the chelating behavior of the following agents were underway: 1,4,7,10-tetraazacyclododecane-*N,N′,N″,N‴*-1,4,7,10 tetraacetic acid (DOTA), 4, 7, 13, 16, 21, 24-hexaoxa-1, 10-diazabicyclo 8.8.8-hexacosane (Kryptofix2.2.2), 5,11,17,23-tetra-*tert*-butyl-25,26,27,28-tetrakis(carboxymethoxy)calix[4] arene-tetraacetic acid (calix[4]-tetraacetic acid), diethylene triamine-*N,N′,N″*-pentaacetic acid (DTPA), 2,2′,2″,2‴-(Ethane-1,2-diyldinitrilo)tetraacetic acid or ethylene diamine tetra acetic acid (EDTA), polyoxopalladates and 18-membered macrocyclic chelator macropa [58,59,63,64].

More recently, in an attempt to overcome the delivery challenge, nanoparticles have become embraced as carriers for radioisotopes. In addition to oncological applications, however, Radium-carrying nanoprobes could also be exploited as antiviral agents [65]. Based on the pioneering results of Pijeira et al., for example, folic acid-functionalized graphene quantum dots (GQD-FA) seem to be useful tools against Zika virus (ZIKV) infection; moreover, the ^223^Ra-labeled radioactive counterpart (^223^Ra@GQD-FA) may also broaden the therapeutic avenue of tumors with folate-receptor upregulation [65].

Figure 2 shows the decay chains of ^223^Ra (Panel A) and ^224^Ra (Panel B).

### 1.3. Nanoparticles for Targeted Radionuclide Delivery

Nanoparticles with an intrinsic ability to transport cancer therapeutics are an emerging category of nanoscale platforms that holds great promise in targeted tumor treatment [66,67,68]. A wide spectrum of nanoparticles is currently available; examples include but are not limited to polymeric nanoparticles, liposomes, micelles, quantum dots, carbon nanotubes, dendrimers, magnetic nanoparticles, and solid lipid nanoparticles [69]. The application of anti-tumor agents packaged into nanocarriers could bypass the limitations of traditional oncological therapies, including unspecific targeting, accidental death of healthy cells, and the development of systemic side effects or drug resistance [70,71]. In addition to chemotherapeutic drugs, small molecule inhibitors, peptides, proteins, nucleic acids (RNAi) and radioisotopes can be loaded into nanoparticles.

Nanomaterial-based cytotoxic medications are reported to be superior to either free drugs or other therapeutic molecular conjugates in several facets [70,72]. First, the attachment of the therapeutic agents to different nanoparticles guarantees more selective transport to target sites coupled with enhanced solubility, bioavailability and tissue permeation that ultimately leads to improved overall anti-tumor therapeutic gain [67,72,73,74]. By choosing the size of the nanoparticle, the tumor retention and therefore the therapeutic effect of the delivered cargo could be flexibly regulated, which constitutes another advantage of the use of nanoparticles as carriers [72,75]. More specific targeting along with lengthened tumor residence time ensure improved killing effect at the disease sites. The size and the shape of the nanoparticles along with the chemical properties of the surface coating are the key determinants of tumor uptake and related therapeutic effect [76,77,78,79]. For this reason, these factors must be taken into account during the de novo construction of nanomaterials to attain a maximal anti-tumor effect with negligible systemic toxicity. Based on the latest literature data, the addition of biocompatible materials onto the surface of the nanomaterials influences their biodistribution and tumor accumulation and therefore the therapeutic efficacy [79,80]. Affecting the interplay between nanoparticles and serum proteins/cell membranes, surfaces charges have a major role in the determination of the organ uptake pattern and the circulatory half-time of the nanomaterials [79,81,82]. In a prior study of Arvizo and co-workers—for example—nanoparticles with neutral (hydroxyl group-terminated, −1.1 mV) and zwitterionic (quaternary ammonium and sulfate group-terminated, −2.0 mV) surface charges appeared to have prolonged blood half-time coupled with higher uptake in the tumor sites [80]. In addition to these, the internalization of polyethylene glycol (PEG) and dextran into the surface of the nanoagents serves as another way to develop highly biocompatible molecules [83,84].

Nevertheless, the facile adjustment of drug extravasation from the nanoconjugates to its mechanism of action also serves the achievement of the required anti-tumor effect [72]. Compared to their free matches, nanocarrier-drug formulations provide more aggravated drug absorption into the tumor, which further contributes to attaining desirable cytotoxicity [70]. In addition—compared to other drug transporters such as antibodies—they have the potential to deliver larger amounts of molecules [85,86] promoting elevated drug concentration levels at the lesions of interest. Importantly, neither the type nor the number of the transported particles modify their organ distribution or pharmacokinetics. Moreover, nanoparticles not only make the delivered drugs resistant against degradation [70], but owing to their endocytosis-mediated cellular entrance, they are not prone to multidrug resistance (MDR) processes, either [72]. In comparison with free therapeutic counterparts, nanocarrier-engulfed drugs are protected even against any interplay with the biological microenvironment [70], which together with the above-listed benefits has a major role in the maximization of the therapeutic effect. By the same token, the nanocarrier coverage promotes drug stability enhancement as well as the extension of the circulatory half-time, thereby aiding the establishment of a favorable safety profile [87,88].

Since the size of the nanoparticles makes the encapsulation of several ligands with distinct receptorial target sites possible, nanomaterials also allow for the development of multivalent delivery systems [89]. Characterized by higher receptor binding affinity, multi-receptor targeted nanosystems can cover an extended subset of cell surface receptors, which apart from the improvement of therapeutic efficacy contributes to better diagnostics during treatment follow-up. In addition, the self-assembly of nanostructures enables the construction of multifunctional nanosystems with improved biocompatibility and biodegradability that could be exploited in targeted anti-cancer or even in gene drug delivery [90,91]. Furthermore, self-assembled nanostructures including nanoparticles, nanotubes, nanofibers, or hydrogels are characterized by adjustable bioactivity, variable surface chemistry, and target specificity that along with the low production costs also make them emerge not only as promising drug carriers but also as useful tools for different biological purposes such as tissue regeneration or bioimaging [92,93]. Since the assembly of separate nanoparticles results in the merge of their inherent properties, the regulation of self-assembly leads to the development of complex nanoplatforms with a wide range of characteristics [94,95,96]. In addition to the regulation of conventional nanoparticle assembly, the control of nanoparticle accumulation and disintegration—denoted as reversible self-assembly—may continue to broaden the horizon of nanomaterial applications in medical fields [94]. Furthermore, bispecific nanosystems make simultaneous target-specific treatment and imaging possible. For example, the use of a magnetic bispecific cell engager (MagBICE)—composed of biodegradable iron nanoparticles and antibodies that join the therapeutic cell to the treated cell (Bispecific antibodies)—combines therapy and magnetic imaging [97,98]. Integrating photodynamic therapy and immunotherapy, NJ nanoparticles (prodrug of NLG919 and JQ1) coated with photosensitizer pyropheophorbide-a (PPa)-modified PHP (PHPNJ) are also promising examples for bispecific nanoparticles [99].

Overall, considering the advanced therapeutic indices and the simultaneous reduction in treatment-related side effects [72] along with the above-detailed encouraging results, the translation of nanosystems into further stages of research seems straightforward.

Despite these beneficial properties, there are some potential downsides and challenges that pose an issue on their routine application. Given the possible toxicity of nanocarriers, the assessment of in vitro and in vivo cytotoxic and immunotoxic activity is a prerequisite for usage [56,100]. Although the evaluation of their in vivo behavior is an area ripe for further research, available data suggest that the physicochemical characteristics of nanomaterials—for example size or surface area—that affect their binding properties to blood components, elimination kinetics and the cross-talk with cellular and endothelial barriers largely determine their in vivo fate. Consequently, it impacts their ability to enter different regions/body compartments, which could be associated with potential toxic effects [72,101]. There are a couple of papers reporting the safety of different NaA nanozeolite nanomolecules including NaA-silane-PEG or its prostate-specific membrane antigen (PSMA) targeting match—NaA-silane-PEG-D2B—in normal prostate cell lines (RWPE-1, HPrEC) and in prostate cancer cell lines (LNCaP C4-2, DU-145) [56,102]. Comparably, no obvious signs of in vitro toxicity were observed in association with several nanoparticles such as nanozeolites A, nanozeolites Y, pure silica nanozeolite silicalite-1, unaltered NaA nanozeolite, and Barium-133-labeled nanozeolite A (BaA) attached to poly(ethylene glycol) (PEG) of long chain (BaA-silane-PEGm(MW1000), BaA-silane-PEGm(MW2000)) [103,104,105]. Based on literature data, toxicogenomics serves as a promising tool to explore nanocarrier-associated immunotoxicity and related processes such as hypersensitivity, immunogenicity, autoimmunity, and immunosuppression [56,106,107]. In contrast to the above-mentioned information, Lankoff et al. [56] could not draw a conclusion regarding the in vitro immunotoxic effects of NaA nanozeolite nanocarriers (NaA-silane-PEG and NaA-silane-PEG-D2B) because they experienced the deregulation of both the pro- and anti-inflammatory genes induced by these nanocarriers [56,108,109]. For this reason, relevant studies are required to uncover those aspects of nanoparticle-related toxicity that are poorly understood yet.

Albeit meaningful progress has been made toward the understanding of how nanoformulations interreact with the environment, the complete discovery of the way these compounds pass through the tumor mass still remains part of future research [72,110]. In this respect, it is therefore worthwhile to continue exploring the role of physiological barriers, tumor heterogeneity and hypoxia, endo-lysosomal escape mechanism, as well as the way of elimination [67] to fully elucidate and improve their mode of action. In addition, to impede too early dissociation of the drug from the nanoconjugates, the optimization of release kinetics should be another important step that would definitely increase the translational potential of nanosystem-based therapeutics to in-human application. Further shortcomings may arise from the costs and technical hurdles of the synthesis of nanomaterials.

Even though the listed uncertainties of nanoparticles need to be addressed, their valuable properties fueled enthusiasm for the construction of nanoformulations capable of targeted radioisotope delivery [43,102,111,112]. As it was confirmed by research findings, Ra was successfully coupled to lanthanum phosphate, nanozeolites, barium sulfate (BaSO_4_), hydroxyapatite, and calcium carbonate (CaCO_3_) nano/microparticles [31,43,61,62,112,113,114,115,116,117,118]. Furthermore, gypsum (CaSO_4_) or celestine (SrSO_4_) also appear to be highly desirable inorganic applicants for the de novo design of Ra-labeled nanomaterials [119]. In addition to these, organic particles—for instance, liposomes—are acknowledged as well-suited nanocarriers to stably join Ra to various targeting compounds [120,121]. As exemplified by earlier studies, the therapeutic value of Ra-loaded nanoparticles was confirmed at a preclinical level [31,43,56,112].

The use of carriers guarantees prolonged residence time for the Ra isotopes in the target location and therefore allow for enhanced therapeutic effect at the disease sites. Thus, they not only serve the purpose of ensuring Ra uptake beyond the skeletal system, but they also have a remarkable contribution to the improvement of anti-tumor radiotherapeutic effect. In addition, microparticles may also aid in reducing the untoward radiation of non-target organs by retaining much of the release of Ra decay products derived from the nuclear recoil effect [60,122].

In this review, we discuss the therapeutic potential of ^223/224^Ra-doped nanoparticles in preclinical models to present an up-to-date overview of recent advances in targeted alpha radiotherapy.

## 2. Overview of In Vivo Results with ^223/224^Ra-Labeled Nanoparticles

Considering the above-mentioned characteristic of Ra radionuclides, the construction of ^223^Ra/^224^Ra-based nanoparticles has attracted the attention of several researchers. Ample amount of preclinical experiments were directed toward the radiosynthesis, the radiochemical analysis and the in vitro characterization of ^223^Ra-labeled nanocarriers [123,124,125,126]. Based on these results, several nanoparticles bound to ^223^Ra seem to be suitable for further in vivo biomedical applications including hydroxyapatite, titanium dioxide (TiO_2_), Fe_3_O_4_ superparamagnetic iron oxide nanoparticles (SPIONs), barium ferrite nanoparticles (BaFeNPs), gadolinium vanadate nanoparticles (GdVO_4_), barium sulfate (BaSO_4_), detonation nanodiamonds (NDs), reduced graphite oxide (rGiO), multi-walled nanotubes (MWCNTs), and radium dichloride nanomicelles ([^223^Ra]-RaCl_2_) [61,123,124,125,126,127,128,129,130]. Experiencing satisfying in vitro stability for [^223^Ra]Fe_3_O_4_ nanoparticles in various biological niche such as PBS, bovine plasma, and serum, Mokhodoeva et al. strengthened their potential for in vivo MRI, nuclear imaging and theranostic purposes [124]. The results of Suchánková et al.—according to which both the intrinsic labeling and the surface labeling of hydroxyapatite and titanium dioxide nanoparticles with ^223^Ra resulted in labeling yields greater than 94%—confirm the use of these nanomaterials as effective radioisotope transporters in in vivo applications [125]. In addition, using HER2 receptor-positive SKOV-3 cells [127], ^223^Ra-labeled BaFeNPs attached to trastuzumab (Herceptin^®^, [^223^Ra]BaFe–CEPA–trastuzumab) exhibited excellent targeting capability, cell internalization and cytotoxicity that projects BaFeNPs as promising radionuclide carriers in nuclear medicine. Moreover, results on the sorption of various radionuclides (^9m^Tc, ^207^Bi/^213^Bi, ^90^Y,^226^Ra/^223^Ra) on different carbon nanomaterials (CNMs: NDs, rGiO and MWCNTs) as well as findings on their controlled release from the CNM surface area indicate the feasibility of CNMs for radiopharmaceutical development [128]. Although further steps need to be taken to enhance radionuclide retention inside the GdVO_4_ NPs, given the partial encapsulation of ^223^Ra (~75%) in GdVO_4_ NPs and its maximum leakage of 73.0 ± 4.0%, GdVO_4_ NPs are also viable alternatives for isotope transport [129]. Finally, the higher cytotoxic effect of [^223^Ra]RaCl_2_ nanomicelles on SAOS2 bone cancer cells in comparison with [^223^Ra]RaCl_2_, warrants a place for nanomicellas regarding targeted radioisotope delivery [130].

### 2.1. ^224^Ra-Labeled Nanomaterials

#### 2.1.1. Preliminary Results with ^224^Ra-Labeled CaCO_3_

Although Ra-labeled nanoparticles are still in their early stages of application in in vivo preclinical imaging, preliminary results indicate great promise [43,56,102]. Table 1 presents an overview of the preclinical studies on Ra-labeled nanoparticles. Investigating the uptake pattern of Radium-224 (^224^Ra), Westrøm et al. performed biodistribution studies with the free cationic form of ^224^Ra (^224^RaCl_2_) and with ^224^Ra-labeled calcium carbonate microparticle (CaCO_3_) suspensions (0.4 mL) of three different particle types in healthy nude mice 1, 4, and 7 days post-intraperitoneal (ip.) tracer administration (as seen in Table 1) [112]. First-generation, second-generation and PlasmaChem microparticles with diameters from 3 to 15 μm, 1 to 3 μm, and 1 to 3 μm, respectively, were prepared by the spontaneous precipitation method [131]. All ip. organs exhibited radioactivity after the injection of the first-generation ^224^Ra-CaCO_3_ including the liver, the stomach, the intestines, the parietal peritoneum, and the ip. fat tissue, except for the spleen, which showed signs of tracer concentration post-^224^RaCl_2_ application (both labeled in Dulbecco’s PBS with 0.5% BSA). Although due to the affinity of ^224^Ra to the bones, the highest free ^224^Ra accretion was present in the skeletal system (skull, femur), the osseous uptake of the first-generation ^224^Ra-CaCO_3_ significantly decreased, which indicated moderate release of the isotope from the microparticle (both labeled in Dulbecco’s PBS with 0.5% BSA). Interestingly, ^224^Ra efflux from the smallest PlasmaChem (labeled in sucrose) microparticles was reported to be more enhanced. The skeletal uptake of ^224^Ra—which is proportional to the release of the isotope from the microparticle—shows negative association with the radioactivity that remained in the peritoneum, which is of central importance in terms of the ip. therapeutic effect of the isotope. Since varying ip. ^224^Ra retention could impact the radiotherapeutic efficacy, appropriate chelation is essential to achieve targeted cytotoxicity.

Of note, faint cardiac, cerebral and muscle activity was displayed for both the free ^224^Ra and the first-generation radiopharmaceuticals (both labeled in Dulbecco’s PBS with 0.5% BSA) [112]. The higher lienal radioactivity for the first generation agent—labeled in sucrose solution—compared to that of the counterpart labeled in Dulbecco’s PBS with 0.5% *BSA* or that of the PlasmaChem could be accountable to the activity of the peritoneal fat measured in the close vicinity of the spleen.

Apart from skeletal uptake, which negatively correlated with the administered particle amount, when looking at additional factors affecting particle uptake such as particle mass (i.e., what else influences particle uptake), the same biodistribution profile was observed when administering 1, 5 and 25 mg of the 2nd generation CaCO_3_ as before. Therefore, the choice of the optimal particle size and the determination of the administered dose are as important as the selection of the appropriate linker for the radioisotope.

#### 2.1.2. In Vivo Evaluation of ^224^Ra-CaCO_3_ in Therapeutic Settings

##### Tumor Growth Inhibition in Preclinical Models of SKOV-3luc Tumors

As a result of these findings, the interest of the same research group turned toward the investigation of ^224^Ra-CaCO_3_ in therapeutic settings [43]. Table 1 summarizes the details of their study. Based on tumor volume reduction or prolonged survival and the lack of meaningful systemic toxicity, Westrøm et al. reported the therapeutic feasibility of alpha-emitter ^224^Ra-labeled CaCO_3_ microparticles in mice bearing intraperitoneal ovarian cancer [43,112]. Applying different treatment regimes, female athymic nude Foxn^nu^ mice ip. inoculated with SKOV-3luc human ovarian epithelial adenocarcinoma cell lines were ip. injected with ^224^Ra-CaCO_3_. Upon the post-treatment measurement of the SKOV3-luc tumors, notable tumor growth inhibition was experienced in the treated cohort compared to the control regardless of the administered dose (65 or 200 or 3 × 65 kBq/BW(kgs)) or the number of treatments (one or three). The results of D-luciferin-based in vivo bioluminescence imaging—showing bioluminescent signal intensity enhancement in the untreated animals relative to the medicalized ones 28 and 45 days after SKOV3-luc tumor induction—were also in accordance with the tumor weight estimations. The elevated pervasiveness of solid tumors in the SKOV3-luc tumor-bearing control mice in comparison with the treated animals further validated the anti-tumor efficacy of the labeled probe.

##### Tumor Growth Inhibition in Preclinical Models of ES-2 Tumors

In addition to SKOV-3-luc tumors, a couple of prior studies reported on the therapeutic efficacy of ^224^Ra-CaCO_3_ in the targeted treatment of ES-2 ovarian tumors [31,43]. Given the lengthened median survival and decreased amount of ascites of the irradiated ES-2-transplanted mice compared to the saline-injected controls, Westrøm and co-workers proved the therapeutic power of ^224^Ra-CaCO_3_ in an ovarian tumor model of more advanced disease stage as well [43]. Upon the ip. administration of four different types of ^224^Ra-CaCO_3_, the same research group led by Napoli managed to achieve similar survival benefit in ES-2 tumor-bearing mice to that of Westrøm and co-workers; moreover, they concluded that the experienced therapeutic success was not dependent on either the labeling method or the addition of polyacrylic acid (PAA) coating to the microparticles [132]. The research of Napoli et al. (2021) [132] is summarized in Table 1.

Similarly to the SKOV3-luc models, Westrøm et al. did not find any significant difference between the survival length of the ES-2 xenotransplanted mice of the different treatment cohorts either (150, 300, 1000, or 2 × 150 kBq/kg); therefore, we may conclude that neither dose increment nor repeated treatments influence the anti-tumor potential of ^224^Ra-CaCO_3_ particles. The advanced stage of the neoplasms, inferior peritoneal dispersion of the ^224^Ra-CaCO_3_ microparticles and their rapid clearance from the abdominal cavity could underpin the lack of dose dependency [43]. Additionally, the loss of p53 protein which has a central contribution to radiation regulation may also be responsible for the lack of the development of dose-dependent radiosensitivity [133,134,135,136]. Transcription factor p53 has a central role in mediating cellular response to radiation [134]. DNA damage induced by radiation exposure triggers the accumulation p53 protein [137,138], which activates cellular pathways related to apoptosis, cell death or DNA repair mechanisms and cell survival [139,140]. Therefore, the loss of p53 or alterations in its mechanism of action may be associated with cancer development and resistance to chemo- and radiotherapy [141,142,143]. Although recent data confirmed the inevitable contribution of p53 to radiation control, its role is largely dependent upon the type of the irradiated organs and cells [140]. In addition to the above-mentioned information, however, microparticle aggregates may preclude alpha particles from being evenly distributed in the peritoneum, which could constrain the effective irradiation of some target tissues and organs [31]. Furthermore, the short-lived nature of ^224^Ra and related shorter tumor retention time may also provide some possible explanations. Correspondingly to the results of Westrøm et al., Larsen and colleagues did not find a relationship between the dose of the administered radioactivity and the therapeutic response either, applying ^211^At-labeled monodisperse polymer particles (^211^At-MDPP) in a preclinical ip. K13 hybridoma model [144]. In their study, identical survival was observed upon the administration of 7, 65, 200 or 500 kBq of ^211^At-MDPP to Female Balb/c mice ip. xenotransplanted with K13 hybridoma cells [144].

Similarly to Westrøm et al., Li and colleagues also assessed the anti-cancer capability of ^224^Ra-CaCO_3_ in ES-2 ovarian tumor models of female athymic nude-Foxn1^nu^ mice (presented in Table 1) [31]. In light of the improved survival, Li et al. further validated the therapeutic success of the isotope-loaded microparticle (0.4 to 4.6 kBq/mg of ^224^Ra-CaCO_3_), which was in corroboration with the findings of Westrøm et al. and Napoli et al. [31,43,132]. Albeit notably lengthened survival was detected in the group administered with the free cationic form of ^224^Ra (^224^RaCl_2_) compared to the untreated animals, the best therapeutic outcome achieved by the carrier-coated probe (^224^Ra-CaCO_3_) indicates the therapy-enhancing effect of the CaCO_3_ microparticle (survival rates in the control, ^224^RaCl_2_, and ^224^Ra-CaCO_3_-treated cohort: 0/9, 1/5, and 10/10, respectively) [31]. In a like manner, earlier findings of Larsen et al. with free ^211^At and ^211^At-MDPP also demonstrated the significance of microparticles in exploiting the radiotherapeutic effect of the transported isotopes [144]. Radionuclides bound to nano/microparticles have extended the residence time in the tumor niche compared to the free matches that is essential for the exertion of sufficient cytotoxic effect. ^224^Ra-CaCO_3_, which requires 25% lower radioactivity, has the same anti-tumor efficacy as ^224^RaCl_2_, which indicates less radiation exposure associated with ^224^Ra-CaCO_3_ handling. All this is of utmost importance regarding its integration into standard patient care [31]. In a similar way, this favorable safety profile was authenticated by Westrøm et al. [43]. We assume that the therapeutic gains of ^224^Ra-CaCO_3_ coupled with the reduced radiation danger will drive its optimization and the continuing investigation into further stages of clinical research prior to being an approved product for cancer patients.

The therapeutic indices measured by Li and colleagues in the low (240–300 kBq/kg), intermediate (380–460 kBq/kg), and high-dose (940–1140 kBq/kg) treatment groups with corresponding values being 1.5, 1.8 and 2.8 suggest a kind of dose–response relationship between the administered radioactivity and the therapeutic outcome [31]. This may correlate with the findings of Westrøm and co-workers, who noted a similar trend; however, no statistically significant direct association was confirmed between the injected dose and the success of the therapy [43]. Varying treatment setups or tumor stages could be supposed as possible explanations for this difference between the two studies. Unlike Westrøm et al., who noted reduced femoral ^224^Ra-CaCO_3_ activity (meaning reduced systemic release) with increasing mass doses that led to improved therapeutic outcome, Li et al. did not point out a similar negative correlation [31,112]. As the skeletal uptake is positively associated with isotope release form the nanoparticle and therefore is inversely proportional to the radiotherapeutic effect at the target site, factors that affect radionuclide efflux—other than mass doses—must be further investigated. Furthermore, the remarkable reduction in ascites volume registered by Li et al. in relation to the treatment active group compared to the controls (mean volume: 1.4 vs. 4.7 in the treated and in the untreated cohorts, respectively) is closely associated with the observations of Westrøm and co-workers [31].

As the diffusion of Thoron/220-Radon (^220^Rn)—one of the decay progenies of ^224^Ra—from the pores [131] of the microparticles, is reported to enhance anti-tumor ability, Napoli et al. aimed to investigate the effect of the way of radiolabeling on ^220^Rn release with ES-2 xenograft models [132]. Applying surface absorption or inclusion-labeled CaCO_3_ microparticles, Napoli et al. proved that the different labeling procedures neither influence the release of ^220^Rn from the microparticles nor the anti-tumor effect of the probes in the ES-2 tumor-carrying mice [132]. Inclusion-labeled ^224^Ra-CaCO_3_ microparticles with a PAA coating, however, were associated with moderately lower anti-tumor potential, which was presumably due to the presence of the polymer layer that led to the decrease in ^220^Rn release from the microparticles [132]. Table 1 gives a brief summary of the most important points of their study.

Based on the findings obtained, it can be established that more consistent irradiation of the peritoneum induced by the efflux of ^220^Rn may aid in overcoming the limitations deriving from inappropriate ^224^Ra-microparticle distribution [132] and subsequent dose inhomogeneity, which could result in remarkable therapeutic enhancement. Furthermore, ^220^Rn emanation can generate the expansion of the penetration depth of alpha particles, making their transport beyond the general alpha particle traveling distance possible. It means that Radon-220 diffusion extends the penetration range of alpha-emitting Radium-224, and this contributes to the therapeutic efficacy of ^224^Ra-labeled nanoparticles. Owing to the 3-fold higher mean diffusion length of ^220^Rn (300–400 μm) compared to the maximum pathway of an alpha particle (100 μm) in water, an extended volume could be irradiated via the release of the first progeny of ^224^Ra—^220^Rn [132]. By the same token, diffusing alpha-emitters radiation therapy (DaRT) that is based on the emission of the alpha-emitting decay products of ^224^Ra is proposed as a novel method of targeted cancer treatment [29,145,146,147]. With the application of ^224^Ra-labeled wires embedded into experimental squamous cell carcinoma tumors, Arazi et al. demonstrated a necrotic area with a diameter of 5–7 mm corresponding to the irradiated region, which supported the anti-tumor efficacy of progeny-related radiation [29]. Accordingly, initial trials also affirmed the therapeutic advantages of the radiation stemming from daughter nuclei in DaRT under clinical circumstances in the treatment of locally advanced and recurrent squamous cell carcinoma (SCC) of the skin and the head and neck [147,148].

To make sure that alpha rays reach the lesions of interest, apart from ^220^Rn efflux, the microparticles must also be localized close to the tumor to be irradiated. This is also of pivotal importance for the maintenance of constant irradiation.

#### 2.1.3. Toxicity Evaluation

The absence of ^224^Ra-CaCO_3_-induced histopathological cytotoxicity coupled with no signs of irreversible, treatment-related hematological and clinical chemical abnormalities project the safety of the compound, which is of utmost importance regarding its transportation to clinical usage [43]. The toxicity profile of ^224^Ra-CaCO_3_ is summarized in Table 2. Correspondingly to the observations of Westrøm et al., neither did Li et al. encounter any acute or subacute toxic effects in association with the ^224^Ra-CaCO_3_ treatment of ES-2 xenotransplanted mice (as shown in Table 2) [31]. On the contrary, another research study of Westrøm and co-workers revealed that ^224^Ra bone uptake is inversely proportional to the injected dose, which indicated the possibility of bone marrow toxicity [112]. In addition to this, toxic effects coming from the redistribution of the decay products of ^224^Ra should also be addressed [132,149,150,151]. Through reporting hepatic and red blood cell ^212^Pb (T_1/2_ = 10.64 h) overaccumulation along with renal ^212^Bi (T_1/2_ = 60.54 min) uptake in beagles administered with ^224^Ra, Lloyd et al. drew the attention to the potential organ toxicity of ^224^Ra [149,152]. In a similar way, progeny-related adverse effects were published by Milenic and co-workers who investigated the acute and long-term effects of ip. or intravenously (iv.) injected ^212^Pb-TCMC-trastuzumab [150]. The relevant findings of their study are displayed in Table 2. Seven or 90 days post-injection of Balb/c mice with ^212^Pb-TCMC-trastuzumab (ip. and iv.: 0.0925–1.85 MBq and 0.0925–1.11 MBq, respectively), ^212^Pb-associated toxicity was observed in the bone marrow, spleen, kidneys, and liver, which was confirmed by histopathological examinations (demonstrated by Table 2) [150]. According to Wick and Gössner’s observation, the radiation of healthy tissues—induced by the release of daughter nuclei—could lead to renal dysfunction and tumor development such as skeletal malignancies or chronic myeloid leukemia. These observations of theirs perfectly correlated with prior findings [153]. By registering more elevated renal ^212^Pb accretion for the first generation ^224^Ra-CaCO_3_ particles than for the free ^224^Ra (dissolved RaCl_2_), Westrøm et al. also corroborated the redispersion of the isotope from the target site to remote organs and tissues [112]. These results highlight the importance of the integration of tissue/organ toxicity assessment in the design of future studies with radiolabeled nanoparticles. In addition to the appearance of unwanted toxicity—given the meaningful contribution of the alpha-decaying nuclides of ^212^Pb to the alpha energy release of ^224^Ra—its reallocation to organs far from the point source may reduce the overall therapeutic dose [132,145,154,155]. Therefore, to mitigate treatment-related adverse effects and to maintain ideal therapeutic outcome, attempts should be made to diminish the efflux of progenies. As part of liquid phase experiments, Napoli et al. noted the adsorption of ^212^Pb by ^224^Ra-CaCO_3_ microparticles, which in part may possibly reduce the redistribution of the daughter radioisotope [132]. In line with this, based on the in vitro retention studies of Westrøm et al., the mean retained ^212^Pb radioactivity was reported to exceed 95% in case of PlasmaChem (5 μm) and first-generation ^224^Ra-CaCO_3_ (18 μm) microparticles that were radiolabeled in sucrose solution and maintained in the same medium [112]. We may conclude that the particle size as well as the way of particle synthesis influence the radioisotope retention of the nanoparticles.

Despite all this, previous in vivo research comparing α-emitter-based ip. treatment with β therapy proved that the former ones performed better regarding anti-tumor and/or toxicity aspects [20,156,157]. Although performed with antibodies as carriers, pioneering clinical trials even demonstrated negligible toxicity in relation to ip.-administered α-radiotherapy [158,159,160].

In contrast to previous preclinical and clinical findings, the lack of treatment-related particle residuum [43] authenticated the biodegradable and biocompatible nature of CaCO_3_ [161,162]. Two weeks post-intracavital administration of 90 kDa poly(lactic-co-glycolic) acid (PLGA) micro, and nanoparticles (5–250 µm) to male SV129 mice, Kohane and colleagues noted a meaningful presence of permanent residue, adhesions, histopathological signs of chronic inflammation and foreign body giant cells [161]. Identically, within the framework of prior organ uptake studies on ^224^Ra-CaCO_3_, microparticle remnants were depicted by Westrøm et al. in immunodeficient healthy female athymic nude Foxn^nu^ mice after the ip. injection of different types of radiolabeled CaCO_3_ microparticles [112]. Based on necropsy experiments, they found a positive correlation between the quantity of the peritoneal leftovers and the amount of the injected microparticles. Remnants smaller than a diameter of 3 mm were related to CaCO_3_ microparticles between 1 and 5 mg, whereas post-injection of microparticles of 25 mg, larger residuum were encountered. The observations of an earlier in-human phase I study with 12 gynecological cancer patients reported polymer residues adhesions, fat necrosis, and foreign body giant cell development in association with the administration of paclitaxel microspheres, which also contradicted the results of Westrøm et al. [43,162].

In addition, we must consider the fact that—like radioactive radiation in general—the positive and negative effects of the therapeutic isotopes depend on the taxonomic classification of the irradiated animal (mouse, rat, dog) and, within that, on the strain and species. It is also known from the literature that within the strains, the radiation effect (toxic, therapeutic) induced by either external or internal radiation sources is greatly influenced by the gender, age, immunological status, repair mechanisms (e.g., DNA repair) as well as the status of the cell cycle and oxygenation of the irradiated tissues, etc. [163,164,165].

#### 2.1.4. Clinically Transportable Findings

Westrøm et al. depicted a positive correlation between the SKOV3-luc tumor bulks and the neutrophil granulocyte-to-lymphocyte ratio (NLR), which may be related to worse survival rates [43,166]. Considering the relationship between elevated NLR in ovarian cancer and increased level of tumor marker CA-125, sizeable amount of ascites, more severe disease stage and poorer treatment response, the follow-up of NLR throughout chemotherapy/radiotherapy should raise attention [166]. Presumably due to progressed disease conditions, notably higher white blood cell (WBC) levels were measured in the control and in the 150 kBq/kg ^224^Ra-CaCO_3_-receiving ES-2 bearing mice, indicating the value of WBC as a prognostic biomarker besides NLR. Therapy-related reduction in ascites volume experienced in mice carrying ES-2 tumors [43] is supposed to be an outstanding therapeutic benefit, since based on prior literature findings, the volume of ascites is regarded as an independent determinant of survival in ovarian cancer [167]. Therefore, we suppose that regular blood count monitoring, in particular leukocytes and their subtypes, as well as the measurement of ascites volume should be part of the management of patients under targeted radiotherapeutic treatment. These parameters would not only provide a representation of the current disease state but also would help to assess the response to therapy that could be used to select those patients who would benefit from further treatment.

### 2.2. ^223^Ra-Labeled Nanomaterials

Aside from ^224^Ra, the formerly detailed favorable characteristics of ^223^Ra render increasing interest for its application in the design of target-specific nanocarriers. As previously mentioned, significant efforts have been made to find the best-fitting nanomaterial or chelator to attach ^223^Ra to target-specific molecules [115,116,120,121,125,126].

#### 2.2.1. In Vitro Results with ^223^Ra-labeled Prostate-Specific Membrane Antigen (PSMA)-Targeting Nanoparticle (^223^RaA-silane-PEG-D2B)

According to the in vitro results of Czerwińska and Lankoff, ^223^Ra-labeled prostate-specific membrane antigen (PSMA)-targeting NaA nanozeolites decorated with anti-PSMA monoclonal antibody D2B (^223^RaA-silane-PEG-D2B) could be potential future candidates for the targeted radio-treatment of PSMA receptor-positive prostate cancer (PCa) patients [56,102]. Table 1 presents a resumé on the study of Czerwińska and Lankoff. In vitro binding studies with ^131^I-PSMA-PEG-silane-NaA, internalization experiments and MTT-assay-based metabolic activity measurements—performed in PSMA-expressing LNCaP C4-2 cells (human lymph node prostate carcinoma cell line) [168] and in PSMA-negative DU-145 cells (human epithelial prostate carcinoma cell line) [169]—demonstrated selective receptor binding, rapid and specific cellular internalization and substantial cytotoxicity in PSMA overexpressing LNCaP C4-2 cells for ^223^RaA-silane-PEG-D2B, and this projected its therapeutic feasibility under in vivo circumstances as well (displayed in Table 1) [102].

The results of the annexin/propidium iodide assay also verified the capability of the PSMA-affine nanocarriers in the induction of cell death by showing an increase in the number of the early apoptotic and late apoptotic/necrotic human primary prostate epithelial cells (HPrEC). In a like manner, the more increased number of caspase 3/7 positive apoptotic HPrEC cells related to the administration of NaA-silane-PEG and NaA-silane-PEG-D2B compared to the control cells receiving PBS further strengthened apoptosis induction caused by these compounds (NaA-silane-PEG: 2.5% vs. 1.4% and NaA-silane-PEG-D2B: 3.7% vs. 1.4% for apoptotic and control cells; respectively) [56].

#### 2.2.2. In Vivo Biodistribution of ^223^RaA-silane-PEG and ^223^RaA-silane-PEG-D2B

Despite the promising in vitro apoptosis induction capability, the considerable activity of ^223^RaA-silane-PEG and ^223^RaA-silane-PEG-D2B in off-target organs such as the liver, lungs, spleen, and the bones of PSMA-positive LNCaP C4-2 tumor bearing mice may hamper the application of these derivatives in imaging settings. Moreover, their negligible tumor accumulation coupled with the meaningful background noise limits the precise delineation of the neoplastic mass from the neighboring healthy organs. Hence, biochemical modifications are required to optimize the pharmacokinetics of the nanoconjugates for diagnostic purposes as well. Even though D2B exerts outstanding PSMA selectivity in vivo [170], the low Enhanced Permeability and Retention effect (EPR) of the antibody-impregnated radioconjugate (^223^RaA-silane-PEG-D2B) may impede optimal tumor accumulation [171]. Similarly, association between the EPR effect and the tumor uptake of different nanoparticles was reported in prior preclinical studies with breast, ovarian, pancreatic cancer, and squamous cell carcinoma xenografts [172,173,174]. Thus, special emphasis should be laid on the enhancement of EPR to prolong tumor retention time and delay nanoparticle leakage from the tumor mass [171,172]. In addition, although ^223^RaA-silane-PEG-D2B with a diameter of 250 nm [82] is fitted to the pore size of the subcutaneously developing LNCaP C4-2 prostate cancer, it shows continuous change during tumor progression that impacts EPR effect and related tumor accumulation [175,176,177]. To enhance EPR and thus the therapeutic efficacy of nanoparticles, the size and shape of the nanoagents along with the chemical properties and charge of the surface coating must be addressed and selected carefully [76,77,78,79,178]. Since the blood perfusion makes a critical contribution to the transport of nanoparticles to tumor sites through the EPR [179], the use of drugs such as angiotensin II for blood flow enhancement in the target area could be a possible solution for the improvement of EPR. Furthermore, adjuvants, inflammatory factors, or antibody photosensitizers seem to be feasible to increase drug transport via altering the EPR effect [180,181,182,183].

The exact mechanism behind the experienced high non-target uptake [56] is not yet fully elucidated; nevertheless, the activity of the hepatic, pulmonary and lienal reticuloendothelial (RES) cells could be supposed in the respective organs. According to prior pharmacokinetic findings, the hydrodynamic size, the water solubility, and the presence of “bio-corona” coverage on the surface of the nanoparticles are the major determinants of the cellular uptake and the organ distribution pattern of nanomaterials [173,184,185,186]. Owing to the adherence of the serum components to the outer layer of the administered nanomolecules, a protein–lipid coat—termed as “bio-corona”—is formed on the surface of the nanoconjugates, which is associated with the nanoparticle size [173,186]. Given the more pronounced phagocytotic activity of RES cells in case of nanomaterials of larger hydrodynamic size, the rapid uptake of ^223^RaA-silane-PEG (approx. 196 nm) and ^223^RaA-silane-PEG-D2B (approx. 250 nm) by the macrophages leads to increased accumulation in the corresponding organs [102,173]. The results of Lankoff et al. could be consistent with those of previous studies that confirmed high concentrations of the subsequent nanocarriers in organs related to the RES system: ^99m^Tc-Fe_3_O_4_-1-PNPs-hEGFR (99m Technetium (^99m^Tc)-labeled poly(ethylene glycol) (PEG)-based polymeric nanocarriers (PNPs) with iron oxide (Fe_3_O_4_) nanoparticles and human epithelial growth factor receptor (hEGFR) and ^177^Lu@Fe_3_O_4_-CEPA or ^177^Lu@Fe_3_O_4_-CEPA-trastuzumab nanoconjugates (^177^Lu-labeled supermagnetic iron oxide nanoparticles attached to human epidermal growth factor receptor type 2 (HER2) antibody trastuzumab) [173,187].

According to existing literature data, the in vivo experienced higher skeletal accumulation of ^223^RaA-silane-PEG and ^223^RaA-silane-PEG-D2B [56] could be primarily attributed to the release of ^223^Ra and its daughter nuclides from the microparticles and their reassimilation to the bones [60,112,188]. As opposed to this, the in vitro observations of the same research group showed insignificant isotope efflux from the investigated nanomolecules in human serum [102] that may be caused by the differences between the in vitro and in vivo experimental circumstances. Since in vitro studies are performed under controlled conditions, they may fail to correctly represent the physiological mechanisms/functions/processes of a living organisms. On the other hand, in vivo investigations are accomplished in whole living organisms that resemble better the characteristics of organs and tissues; hence, they supply more reliable results on functioning. Therefore, the results of in vitro studies may not correspond to those of the in vivo ones. In addition, the other free isotope of radium in the form of ^223^RaCl_2_ exhibited lower skeletal uptake compared to the detailed nanoconjugates with greater particle size (^223^Ra-silane-PEG-D2B and ^223^Ra-silane-PEG), which could possibly be attributed to the shorter residence time of ^223^Ra in the bones due its smaller size as well as the reallocation of the radionuclide from the mentioned nanoparticles (Alpharadin (Xofigo) Assessment Report; 2021).

#### 2.2.3. Toxicity Profile of ^223^Ra-Labeled Prostate Cancer-Targeting Nanoparticles

In line with the experience of Li et al., Lankoff and co-workers did not find evidence for therapy-triggered body weight loss either one day after the administration of the unlabeled nanocarriers (NaA-silane-PEG and NaA-silane-PEG-D2B) and the radioconjugates (^223^RaA-silane-PEG and ^223^RaA-silane-PEG-D2B) into healthy BALB/c mice. This indicated the lack of treatment-related acute or subacute toxicity (presented in Table 1) [56]. Although similarly to the findings of Westrøm et al. and Li et al., no signs of treatment-associated toxicity were noted 24 h post-injection of either ^223^RaA-silane-PEG, ^223^RaA-silane-PEG-D2B or their unlabeled derivatives during the follow-up of body weight, various laboratory parameters and histopathological specimens, a week after treatment with the ^223^Ra-labeled compounds, bone marrow fibrosis and a meaningful decrease in the platelet counts and body weight reduction were found in the irradiated study mice [31,43]. The hematotoxicity of ^223^RaA-silane-PEG and ^223^RaA-silane-PEG-D2B was further validated upon the comparison of the one-day and seven-day blood count results, which revealed a significantly deteriorating blood count profile regarding WBC, red blood cell and platelet counts, hematocrit and hemoglobin concentrations as the therapy progressed [56,102]. Consistent with the findings of Lankoff et al., in the phase II clinical trial of Hofman and colleagues, grade 3-4 thrombocytopenia was observed in 27% of castration-resistant prostate cancer (CRPC) patients administered with another PSMA-targeting radioprobe (Lutetium-177 (^177^Lu)-PSMA) [189]. In addition, several clinical studies also revealed comparable hematological toxicity related to targeted ^177^Lu-PSMA-617 treatment in prostate cancer sufferers [190,191,192,193]. In contrast, Westrøm et al. showed no treatment-induced hematological toxicity even 44–45 days after ip. ^223^Ra-CaCO_3_ treatment in SKOV3 tumor-bearing mice (day 0: inoculation; day 3: treatment; day 47–48: blood sampling); however, in the ES-2 xenotransplanted cohort, a discrete platelet number reduction was encountered post-^223^Ra-CaCO_3_ administration that was not statistically meaningful. Based on these observations, we may conclude that treatment with radium-labeled nanoparticles may affect hematopoiesis; hence, the monitoring of blood counts is recommended during such therapies. Emphasis must be placed on the fact; however, that while Czerwińska and Lankoff performed toxicity assessment in a group of healthy mice, Westrøm et al. used tumor-bearing animals for the experiments, which may raise some difficulties regarding the direct comparison of the results of the two studies. In addition, biochemical analyses showed elevated levels of aspartate aminotransferase (AST) and alanine aminotransferase (ALT) for all nanoconjugates (NaA-silane-PEG, NaA-silane-PEG-D2B, ^223^RaA-silane-PEG and ^223^RaA-silane-PEG-D2B) regardless of the presence of radiolabeling [56], which did not match the results of Westrøm et al. and therefore was probably not ^223^Ra-irradiation-related but rather due to the hepatic accretion of the compounds. Identically, in the study of Sun et al., Sprague–Dawley rats intratracheally administered with silica nanoparticles (SiNPs) also exhibited more increased ALT and AST serum concentrations compared to the untreated animal group [194].

#### 2.2.4. Alpha Targeting of Prostate Cancer beyond 223-Radium (^223^Ra)

Currently, apart from ^223^RaCl_2_, there is no other molecule in clinical usage for the targeted therapy of mCRPC [195]. In addition to ^223^Ra, the anti-tumor potential of other alpha emitters including ^225^Ac has also been explored in the treatment of mCRPC patients [196]. Based on the drop of prostate-specific membrane antigen (PSA) levels, and complete therapeutic response on [^68^Ga]Ga-PSMA-11 PET/CT scans, Kratochwil et al. reported the efficacy of ^225^Ac-labeled PSMA small molecule inhibitor PSMA-617 ([^225^Ac]Ac-PSMA-617) in patients with an aggressive type of PCa [193]. Identically, Sathekge et al. experienced an outstanding reduction in PSA concentrations (from 237 to 43 μg/L) after the application of two series of [^213^Bi]Bi-PSMA-617; however, unlike [^225^Ac]Ac-PSMA-617, no treatment-induced adverse health ramifications were perceptible [197].

Although further modifications are required for pharmacokinetical optimization, the results of Czerwińska and Lankoff provided a strong and scientifically justifiable rationale for the future integration of ^223^RaA-silane-PEG and ^223^RaA-silane-PEG-D2B into the treatment armamentarium of PCa.

## 3. 223-Radium (^223^Ra)-Labeled Liposomes

Given the in vivo stability and the favorable organ uptake pattern of ^223^Ra-labeled liposomes, these nanomaterials could also serve as useful tools for the targeted transport of ^223^Ra in therapeutic settings [121]. Upon the biodistribution assessment of ^223^Ra-coated pegylated liposomal doxorubicin (^223^Ra-PLD) in healthy Balb/C mice, Jonasdottir et al. found the highest tracer concentration in the liver and in the spleen, which—similarly to ^223^RaA-silane-PEG and ^223^RaA-silane-PEG-D2B [56]—could have been caused by the phagocytic activity of the RES cells. Table 1 provides a brief overview of the study of Jonasdottir et al. According to initial ex vivo findings, the splenic radiotracer accretion in laboratory mice was higher compared to that of the dogs [121]. The slow blood clearance of ^223^Ra-PLD was evidenced by meaningful blood activities from the early time points (1 and 24 h) as well as the presence of relatively low tracer concentration in the kidneys [121]. In line with this, extremely high liposomal ^223^Ra localization indices (LIs) were registered for the blood, which also supported the prolonged and even the much slower circulatory clearance of the nanoparticle compared to free ^223^Ra. The blood half-life of the liposome-attached ^223^Ra was reported to be more than 24 h. It was in correlation with that of Caelyxì/Doxilì liposome formulation in rodents (24–35 h) and in dogs (23–27 h) [198], whereas the half-life of dissolved ^223^RaCl_2_ in the blood was significantly lower: approximately an hour. Contrary to this, Westrøm et al. did not encounter any differences between the blood elimination of free ^224^Ra and that of the ^224^Ra nanoconjugate (^224^Ra-CoCO_3_) [112]. In agreement with the previous results of Westrøm et al. and Lankoff et al. [56,112], the redistribution of ^223^Ra to the bones from the radioactive liposome (^223^Ra-PLD) could be responsible for the experienced increased femoral and cranial radiotracer uptake [121]. Although recent studies demonstrated that bones are resistant to ^223^Ra-induced bone marrow toxicity to an unexpectedly high extent [47,199], given the long circulatory half-life of the radioactive liposomes along with the large number of nanoparticles in the blood, the potential occurrence of skeletal cytotoxicity must be expected. This finding indicates the importance of the evaluation of bone marrow toxicity and the tracking of related hematological laboratory parameters during in vivo preclinical treatment with liposomal ^223^Ra or even in human application. In addition, dosimetry estimations showing the highest radiation absorbed doses in the spleen followed by the femur, the skull, the kidneys, the lungs, and the skin draw the attention to the potential organ toxicity of ^223^Ra-PLD [121]. Therefore, to avoid unwanted radiation exposure, special emphasis must be placed on the protection of healthy organs and tissues—in particular those of the RES system. Although prior studies reported that the administration of isotope-free liposomes prior to the application of the radioactive counterpart appears to decrease the radioactivity of the RES organs and related radiation risk [200], Jonasdottir et al. had contradictory findings, as they experienced the most elevated liposomal radioactivity in the spleen even after pretreatment with unlabeled doxorubicin liposomes (8.1 mg/kg) [121].

In accordance with previous research that confirmed the therapeutic benefit of liposomes loaded with chemotherapeutic drugs [201], we can conclude that alpha emitter-labeled liposomes either with or without anti-tumor agents may have a place in oncological treatment. However, the physicochemical characteristics and the decay characteristics of the labeling isotope should be carefully considered. Compared to other long-lived alpha emitters such as ^225^Ac (T_1/2_ = 10.0 days) or ^224^Ra (T_1/2_ = 3.6 days), ^223^Ra (T_1/2_ = 11.4 days) seems to be a better applicant for liposome-based drug delivery. This is because considering the half-lives of their progenies, in case of ^223^Ra the first three, while as for the former two radioisotopes, only the first alpha decay occur in the close proximity of the liposome. It obviously has a major role in the achievement of adequate therapeutic effect as well as in the development of unintended radiation side effects. In addition, to prevent potential leakage from the surface of the liposome and related irradiation of healthy organs/tissues, the development of stable liposomes is another issue of concern. In addition to the integration of biopolymers into the liposome structure [202], the encapsulation of liposomes in nanofibers, hydrogels or films [203,204] also serves to enhance the stability of these nanostructures. Aside from these modifications of liposome preparation, different postprocessing techniques such as freeze drying, spray drying and spray freeze drying appear to be valuable methods for stability enhancement [205]. Such postprocessing techniques lead to the development of highly stable dried nanomaterials that could be easily used for drug loading and administration [206].

## 4. Other Alpha-Emitting Radionanoprobes for Intraperitoneal Application

In addition to the information detailed above, several other in vivo studies focused on the investigation of ip. nano/microparticle-based therapy with alpha emitters other than ^223/224^Ra such as ^211^At, ^212^Pb, ^225^Ac or ^227^Th [144,157,207,208]. Since the half-life of these isotopes is the major determinant of their peritoneal retention and thus radiotherapeutic effect, the choice of an α-emitter with a suitable lifespan is mandatory for successful anti-tumor therapy [31]. Similarly to ^224^Ra-CaCO_3_, ^225^Ac, or ^227^Th-labeled anti-HER2 antibody trastuzumab induced prolonged survival in in vivo ovarian or ip. tumor models [209,210,211]. Although identical anti-tumor effect could be achieved applying comparable amounts of radioactivities of ^225^Ac-trastuzmumab, ^227^Th-trastuzumab and ^224^Ra-CaCO_3_, given the long-lived nature of ^225^Ac (T_1/2_: 9.920 (3) days) and ^227^Th (T_1/2_: 18.7 days), targeted radiotherapy with ^224^Ra (T_1/2_: 3.631 (2) days) seems to be more successful as well as more favorable in terms of radiation safety protection [36,43,212,213].

Apart from ovarian tumors, exhaustive research has been centered around the performance evaluation of alpha-emitter radioprobes in the treatment of various ip. tumors [211,214,215]. In comparison with other alpha-emitter therapeutic nanoparticles, including ^213^Bi-Anti-hCD138 monoclonal antibody (mAB), ^213^Bi-labeled humanized domain-deleted mAB (HuCC49DeltaCH2), ^213^Bi-trastuzumab, ^211^At-labeled mAb MX35 (^211^At-MX35), ^211^At-labeled trastuzumab, ^212^Pb-labeled Herceptin, ^212^Pb-labeled 35A7 (anti-CEA) mAB, ^212^Pb-labeled trastuzumab (anti-HER2) mAb, ^212^Pb-labeled anti-epidermal growth factor receptor (EGFR) panitumumab, and B7-H3 epitope targeting 376.96 mAb labeled with ^212^Pb, considerably lower amounts of ^224^Ra radioactivity were enough for the achievement of the same cytotoxic effect in preclinical ip. tumors than in case of the formerly listed probes, and this observation constitutes another clear advantage of the application of ^224^Ra-labeled CaCO_3_ in targeted therapeutic settings [211,214,215,216,217,218,219,220,221].

Due to the greater penetrating power of β emission (few millimeters) relative to alpha (40–80 µm, approximately 2–10 cell diameter), β-emitter therapeutic radionuclides are supposed to exert a cytotoxic effect even in deep neoplastic tissue layers; however, prior research did not strengthen the superiority of ip. β-therapy to alpha radiation [157,222,223,224]. Comparing the therapeutic efficacy of alpha-emitter ^211^At-labeled polymer microspheres (^211^At-microspheres) to that of ^32^P-chromic phosphate colloid or ^90^Y-silicate colloid with beta radiation in K13 hybridoma tumor-carrying female Balb/c mice, Vergote et al. reported improved survival and the highest cure rates in the ^211^At-treated cohort in comparison with the other two treatment active and the control groups [157].

## 5. Highlights for De Novo Nanoradiopharmaceutical Design

Considering the findings of the prior studies on intracavitary treatment with ^224^Ra-nanoparticles, we can summarize that the overall therapeutic outcome is determined by several factors including the injected activity, the specific activity, ^224^Ra retention, ^220^Rn release as well as the re-adsorption of ^212^Pb [31,43,132]. ^220^Rn diffusion may serve to compensate for the downsides of heterogenous microparticle accumulation and related inhomogeneous dose dispersion, while the readsorption of ^212^Pb prohibits its delivery to non-target organs. These decay phenomena simultaneously favor the achievement of improved therapeutic effect. Taking these thoughts into account can aid in continuing the establishment of nanosystems capable of a more precise delivery of drug payloads. 

## 6. Conclusions, Therapeutic Gains form Alpha-Nanoradiopharmaceuticals

Overall, the favorable physicochemical properties of alpha-nanocarriers coupled with their anti-tumor therapeutic benefit confirm the translational potential of alpha nanoplatforms into clinical settings. Targeted radiotherapy with alpha emitters is superior to the other types of radiation in several facets. A reduced incidence of microparticle aggregates in association with alpha therapy ensures a more homogeneous uptake and related uniform dose dispersion compared to other targeted radiotherapeutic treatments, and this in turn favors the preferable application of alpha-radio-nanoparticles [225,226]. Macroaggregates—generating the self-absorption of the alpha particles into the microparticles as well as off-target tissues and organs—not only reduce the therapeutic effect but also contribute to the development of therapy-associated radiation toxicity. In addition, the five-times-higher relative biological effectiveness (RBE) of alpha radiation compared to beta emission also guarantees the therapeutic success of α-nanoparticles [50]. Moreover, due to the short tissue penetration, the use of alpha emitters may yield less toxicity to off-target healthy organs and tissues [227,228]. This short tissue range along with high LET values make alpha emitters more successful in the treatment of micrometastatic lesions compared to beta-emitting radionuclides [229]. Additionally, alpha therapy ensures sufficient dose delivery to the target site presumably due to the high LET of the alpha particles.

With the enhancement of tumor residence time, nanocarriers have a notable contribution to the augmentation of local radiotoxicity [75,230]. Although the application of α-nanotherapy seems to counteract the limitations of β emitters, some downsides of α particles should be carefully considered as well. Given the short penetrating power of α-particles, however, the treatment of sizeable tumor masses brings new challenges and obstacles. Therefore, to ensure therapeutic efficacy, debulking surgery is recommended for tumor mass reduction prior to alpha-radiation [43,231]. High production costs as well as the lack of sufficient isotope supply limit the integration of alpha emitters into both preclinical research and clinical applications [232,233]. Radiation caused by the daughter radionuclides of the alpha emitters is also a matter of concern and must be handled to impede untoward radiotoxicity of tumor-naïve healthy organs [60].

These days, the focus is centered around imaging diagnostics within the field of nuclear medicine; however, due to the wide availability of alpha and beta emitting isotopes, targeted treatment procedures using radionuclides are demanding more and more space. Owing to the continuous development of the physical technology, radiochemistry, pharmaceutical technology and molecular biology, the number of target-specific alpha-emitting radioisotopes (^225^Ac, ^213^Bi, ^224^Ra, ^212^Pb, ^227^Th, ^223^Ra, ^211^At, and ^149^Tb) is steadily increasing in the preclinical, and hopefully—later on—in the clinical practice as well [38]. Beyond the novel and promising radionuclides that are still in their early stage of research, the already well-known and broadly applied Radium isotopes continue to play a key role in one of the newest fields of biotechnology, in nanotechnology.

Overall, the promising results obtained from the studies discussed above demonstrate that the use of nanotechnology in transforming alpha therapy in targeted cancer treatment is the next step forward in today’s research. We expect to see more radiolabeled nanoparticles to be established for therapeutic purposes; for the construction of pharmacodynamically optimized candidates, comprehensive future work is required.

## Figures and Tables

**Table 1:** Overview of the preclinical studies on Ra-labeled nanoparticles.

Reference	Experimental Animals/Cells	Investigated Nanoparticle	Labeling Technique	Investigated Phenomenon	Dosing Regimes	Methods
Jonasdottir et al. [121]	male and female healthy white Balb/C mice	^223^Ra-PLD (80 nm), free cationic ^223^Ra (dissolved ^223^RaCl_2_)	ionophore-mediated loading	in vivo behavior, dose estimation, biodistribution comparison of liposomally encapsulated ^223^Ra and free ^223^Ra	375 kBq/kg (single i.v.)	biodistribution studies (pretreatment with doxorubicin liposomes + treatment with liposomal ^223^Ra, or treatment with solo liposomal ^223^Ra or free ^223^Ra), evaluation of the distribution of the progenies, blood pool clearance measurements, liposomal ^223^Ra dosimetry
Westrøm et al. [43]	SKOV3-luc tumor-bearing female athymic nude Foxn^nu^ mice (SKOV3-luc model of intraperitoneal micrometastasis) ES-2 tumor-carrying female athymic nude Foxn^nu^ mice (ES-2 ascites model)	^224^Ra-CaCo_3_ microparticles (3–15 μm)	spontaneous precipitation method	anti-tumor potential, therapeutic efficacy	65 kBq/kg, 200 kBq/kg, or three injections of 65 kBq/kg (i.p.)	therapeutic efficacy studies: D-luciferin-based bioluminescence imaging, visual tumor inspection, determination of tumor and ascites volume, and body weight Toxicity assessment: histopathological examination of tumor growth, laboratory examinations (hematology, clinical chemistry)
Westrøm et al. [112]	healthy female Athymic nude Foxn^nu^ mice	First-generation (3–15 μm), second-generation (1–3 μm) and PlasmaChem (1–3 μm) ^224^Ra-CaCo_3_ microparticles, ^224^Ra in form of free cation (^224^RaCl_2_)	spontaneous precipitation method	organ distribution with microparticles of different size and amount	single i.p. injection of 2, 6–9, 12, 16 or 22 kBq of ^224^Ra-labeled CaCO_3_ microparticles	microparticle characterization, in vivo and in vitro stability, in vivo biodistribution studies: examination of the effect of microparticle size 1-, 4-, and 7-days post injection, and that of the amount of the microparticles 1 day post injection (1, 5, 25 mg)
Li et al. [31]	female athymic nude-Foxn1^nu^ mice-bearing ES-2 tumors	^224^Ra-CaCo_3_ microparticles (4 to 7 μm), ^224^Ra in form of free cation (^224^RaCl_2_)	precipitation	therapeutic effects (specific activity of ^224^Ra-CaCo_3_)	240–1360 kBq/kg body weight (single i.p.)	Therapeutic efficacy studies: Comparison of ^224^RaCl_2_ and ^224^Ra-CaCO_3_; assessment of the effect of specific activity of ^224^Ra-CaCO_3_ by varying the mass dose of CaCO_3_ as well as the activity dose, measurement of survival time, TI, body weight and ascites volume
Napoli et al. [132]	female Athymic Nude-Foxn1^nu^ mice xenotrasnplanted with ES-2 tumors	surface- and inclusion-labeled ^224^Ra-CaCO_3_ microparticles with or without PAA coating (3–7 μm)	surface adsorption, inclusion labeling of microparticles	therapeutic performance evaluation of the four ^224^Ra-CaCO_3_ microparticles, (diffusion of ^220^Rn from the microparticles, the pathway of ^212^Pb)	138, 179, 350 and 474 kBq/kg body weight (single i.p.)	Therapeutic studies: assessment and comparison of therapeutic effects, follow-up of body weight, and ascites volume (gamma-ray spectroscopy, evaluation of the release of ^220^Rn)
Czerwińska et al. [102]	in vitro: normal RWPE-1 and HPrEC cells, LNCaP C4-2 and DU-145 prostate cells in vivo: healthy BALB/c male mice and LNCaP C4-2 xenograftedBALB/c nude male mice	^223^Ra-labeled, PSMA-targeted NaA nanozeolites (average nominal and hydrodynamic diameter of ~120 nm, and ~200 nm; respectively) ^223^RaA-silane-PEG-D2B and its derivatives: NaA-silane-PEG, NaA-silane-PEG-D2B; ^223^RaA-silane-PEG, ^133^Ba-labeled counterparts: ^133^BaA-silane-PEG and ^133^BaA-silane-PEG-D2B in BA	exchange in Na^+^ for ^223^Ra^2+^ cations in RaCl_2_ solution, ion exchange process	synthesis process of the radioconjugates, in vitro characterization, assessment of in vivo organ distribution, therapeutic efficacy, and toxicity	38, 48, 49, 51 kBq and 185–209 kBq (single i.v.) Average dose: 8.0 ± 1.5–12.6 ± 1.5 mcg kg^−1^ body weight	in vitro studies: assessment of physicochemical properties and stability (*HR-SEM*, *TEM*, *XRD*, *FTIR*, *EDS*, *NTA*, *DLS*, *BET*, *TGA*); assessment of binding specificity, internalization properties, and cytotoxicity, metabolic activity measurement (MTT assay), investigation of cell death (annexin/propidium iodide assay), caspase 3/7 green flow cytometry assay- based apoptosis assessment, gene expression profiling (real-time PCR) in vivo therapeutic studies (^133^BaA-silane-PEG, ^133^BaA-silane-PEG-D2B, PBS, NaA-silane-PEG NaA-silane-PEG-D2B, ^223^RaA-silane-PEG, ^223^RaA-silane-PEG-D2B), in vivo pharmacokinetic and biodistribution studies with ^133^BaA-silane-PEG, ^133^BaA-silane-PEG-D2B, ^223^RaA-silane-PEG, and ^223^RaA-silane-PEG-D2B (%ID g^−1^), toxicity evaluation of ^223^RaA-silane-PEG, and ^223^RaA-silane-PEG-D2B (histopathology H&E, body weight, laboratory tests)

^133^Ba: Barium-133; BET: Brunauer, Emmett, and Teller Measurements; CaCO_3_: calcium–carbonate; D2B: anti-prostate-specific membrane antigen (PSMA) antibody; DLS: dynamic light scattering; DU-145: human epithelial prostate carcinoma cell line (isolated from the brain of a man with prostate cancer); EDF: energy-dispersive X-ray spectrometry; ES-2: human ovarian epithelial adenocarcinoma cell lines; FTIR: Fourier Transform Infrared Spectroscopy; H&E: hematoxylin–eosin; HPrEC: human primary prostate epithelial cells; HR-SEM: High-Resolution Scanning Electron Microscopy; LNCaP C4-2: human lymph node prostate carcinoma cell line; MTT: 3-(4,5-dimethyl-2-thiazolyl)-2,5-diphenyl-2H-tetrazolium bromide; NaA: nanozeolite nanocarriers; NTA: nanoparticle-tracking analysis; PAA: polyacrylic acid; PBS: phosphate-buffered saline; ^212^Pb: Lead-212; PCR: polymerase chain reaction; PLD: pegylated liposomal doxorubicin; PSMA: prostate-specific membrane antigen; ^224^Ra: Radium-224; ^220^Ra: Radium-220; ^224^RaCl_2_: radium-chloride; SKOV3-luc: human ovarian epithelial adenocarcinoma cell lines; TEM: transmission electron microscopy; TGA: thermogravimetric analysis; TI: therapeutic index (median survival time of the treatment group divided by that of the vehicle control); XRD: X-ray diffraction.

**Table 2:** Toxicity evaluation of the assessed nanoparticles.

Radiotracer	Groups	Treatment	Investigated Parameters	Notable Toxicities	Ref.
^212^Pb and ^212^Pb-TCMC-trastuzumab	Healthy mice. Acute toxicity (7 days) and chronic toxicity (90 days)	intraperitoneal (i.p. 0.0925–1.850 MBq) or intravenous (i.v. 0.0925–1.110 MBq) injection into mice	hematology analyses, clinical chemistry, body weight measurement, and histology	Treatment-related effects were observed in bone marrow, spleen, kidneys, liver; Histological alterations: from mild to moderate, indicating low-grade toxicity, and not considered severe enough to affect function; body weight loss in some cases	[150]
^224^Ra-CaCO_3_	Tumor-bearing mice: SKOV3-luc study	i.p. injections of ^224^Ra-labeled CaCO_3_ microparticles with activities of 65 kBq/BW(kgs), 200 BW(kgs), or three injections of 65 kBq/BW(kgs)	hematology analyses, clinical chemistry, and histology	Highest dose group: the liver contained groups of “foamy cells” that may reflect hepatocyte degeneration and/or macrophage-rich foci; Repeated treatment: the liver showed inflammation in portal tracts and piecemeal necrosis of adjoining hepatocytes, The kidney contained tubules with cellular debris of the neutrophil granulocyte type, reflecting acute inflammation	[43]
	Tumor-bearing mice: ES-2 study	i.p. injections of ^224^Ra-labeled CaCO_3_ microparticles: 150 kBq/BW(kgs), 300 kBq/BW(kgs), 1000 kBq/BW(kgs), or two injections of 150 kBq/BW(kgs) into mice	hematology analyses, clinical chemistry, and histology	No toxic effect	
^224^Ra-CaCO3-MP	Tumor-bearing mice: ES-2 study	i.p. injections of ^224^Ra-labeled CaCO_3_ microparticles: 240–1360 kBq/BW(kgs)	body weight measurement	Weight loss was not observed following the treatments	[31]

^212^Pb: Lead-212, TCMC: 1,4,7,10-tetra-(2-carbamoyl methyl)-cyclododecane, i.p.: intraperitoneal, i.v.: intravenous, SKOV-3luc human ovarian epithelial adenocarcinoma cell lines, ES-2: human ovarian epithelial adenocarcinoma cell lines, BW: body weight, MP: microparticle.

## Data Availability

The datasets used and/or analyzed during the current study are available from the corresponding author upon reasonable request.

## References

[1] Barnett G.C., West C.M., Dunning A.M., Elliott R.M., Coles C.E., Pharoah P.D., Burnet N.G. (2009). Normal tissue reactions to radiotherapy: Towards tailoring treatment dose by genotype. Nat. Rev. Cancer.

[2] Koka K., Verma A., Dwarakanath B.S., Papineni R.V.L. (2022). Technological Advancements in External Beam Radiation Therapy (EBRT): An Indispensable Tool for Cancer Treatment. Cancer Manag. Res..

[3] Prasanna P.G., Stone H.B., Wong R.S., Capala J., Bernhard E.J., Vikram B., Coleman C.N. (2012). Normal tissue protection for improving radiotherapy: Where are the Gaps. Transl. Cancer Res..

[4] Marcu L., Bezak E., Allen B.J. (2018). Global comparison of targeted alpha vs targeted beta therapy for cancer: In vitro, in vivo and clinical trials. Crit. Rev. Oncol. Hematol..

[5] Allen B. (2013). Systemic targeted alpha radiotherapy for cancer. J. Biomed. Phys. Eng..

[6] Nikitaki Z., Nikolov V., Mavragani I.V., Mladenov E., Mangelis A., Laskaratou D.A., Fragkoulis G.I., Hellweg C.E., Martin O.A., Emfietzoglou D. (2016). Measurement of complex DNA damage induction and repair in human cellular systems after exposure to ionizing radiations of varying linear energy transfer (LET). Free Radic. Res..

[7] Schumann S., Eberlein U., Muhtadi R., Lassmann M., Scherthan H. (2018). DNA damage in leukocytes after internal ex-vivo irradiation of blood with the α-emitter Ra-223. Sci. Rep..

[8] Pouget J.P., Navarro-Teulon I., Bardiès M., Chouin N., Cartron G., Pèlegrin A., Azria D. (2011). Clinical radioimmunotherapy—The role of radiobiology. Nat. Rev. Clin. Oncol..

[9] Lejeune P., Cruciani V., Berg-Larsen A., Schlicker A., Mobergslien A., Bartnitzky L., Berndt S., Zitzmann-Kolbe S., Kamfenkel C., Stargard S. (2021). Immunostimulatory effects of targeted thorium-227 conjugates as single agent and in combination with anti-PD-L1 therapy. J. Immunother. Cancer.

[10] Leung C.N., Canter B.S., Rajon D., Bäck T.A., Fritton J.C., Azzam E.I., Howell R.W. (2020). Dose-Dependent Growth Delay of Breast Cancer Xenografts in the Bone Marrow of Mice Treated with 223Ra: The Role of Bystander Effects and Their Potential for Therapy. J. Nucl. Med..

[11] Pouget J.P., Constanzo J. (2021). Revisiting the Radiobiology of Targeted Alpha Therapy. Front. Med..

[12] Dekempeneer Y., Keyaerts M., Krasniqi A., Puttemans J., Muyldermans S., Lahoutte T., D’huyvetter M., Devoogdt N. (2016). Targeted alpha therapy using short-lived alpha-particles and the promise of nanobodies as targeting vehicle. Expert Opin. Biol. Ther..

[13] Hoppenz P., Els-Heindl S., Beck-Sickinger A.G. (2020). Peptide-Drug Conjugates and Their Targets in Advanced Cancer Therapies. Front. Chem..

[14] Pouget J.P., Lozza C., Deshayes E., Boudousq V., Navarro-Teulon I. (2015). Introduction to radiobiology of targeted radionuclide therapy. Front. Med..

[15] Sgouros G., Bodei L., McDevitt M.R., Nedrow J.R. (2020). Radiopharmaceutical therapy in cancer: Clinical advances and challenges. Nat. Rev. Drug Discov..

[16] Pallares R.M., Abergel R.J. (2022). Development of radiopharmaceuticals for targeted alpha therapy: Where do we stand. Front. Med..

[17] Wulbrand C., Seidl C., Gaertner F.C., Bruchertseifer F., Morgenstern A., Essler M., Senekowitsch-Schmidtke R. (2013). Alpha-particle emitting 213Bi-anti-EGFR immunoconjugates eradicate tumor cells independent of oxygenation. PLoS ONE.

[18] Allen B.J., Rizvi S.M., Tian Z. (2001). Preclinical targeted alpha therapy for subcutaneous melanoma. Melanoma Res..

[19] Banerjee S.R., Minn I., Kumar V., Josefsson A., Lisok A., Brummet M., Chen J., Kiess A.P., Baidoo K., Brayton C. (2020). Preclinical Evaluation of 203/212Pb-Labeled Low-Molecular-Weight Compounds for Targeted Radiopharmaceutical Therapy of Prostate Cancer. J. Nucl. Med..

[20] Bloomer W.D., McLaughlin W.H., Lambrecht R.M., Atcher R.W., Mirzadeh S., Madara J.L., Milius R.A., Zalutsky M.R., Adelstein S.J., Wolf A.P. (1984). 211At radiocolloid therapy: Further observations and comparison with radiocolloids of 32P, 165Dy, and 90Y. Int. J. Radiat. Oncol. Biol. Phys..

[21] Jurcic J.G., Caron P.C., Nikula T.K., Papadopoulos E.B., Finn R.D., Gansow O.A., Miller W.H., Geerlings M.W., Warrell R.P., Larson S.M. (1995). Radiolabeled anti-CD33 monoclonal antibody M195 for myeloid leukemias. Cancer Res..

[22] Kiess A.P., Minn I., Vaidyanathan G., Hobbs R.F., Josefsson A., Shen C., Brummet M., Chen Y., Choi J., Koumarianou E. (2016). (2S)-2-(3-(1-Carboxy-5-(4-211At-Astatobenzamido)Pentyl)Ureido)-Pentanedioic Acid for PSMA-Targeted α-Particle Radiopharmaceutical Therapy. J. Nucl. Med..

[23] Juzeniene A., Bernoulli J., Suominen M., Halleen J., Larsen R.H. (2018). Antitumor Activity of Novel Bone-seeking, α-emitting 224Ra-solution in a Breast Cancer Skeletal Metastases Model. Anticancer Res..

[24] Morris M.J., Corey E., Guise T.A., Gulley J.L., Kevin Kelly W., Quinn D.I., Scholz A., Sgouros G. (2019). Radium-223 mechanism of action: Implications for use in treatment combinations. Nat. Rev. Urol..

[25] Takalkar A., Adams S., Subbiah V. (2014). Radium-223 dichloride bone-targeted alpha particle therapy for hormone-refractory breast cancer metastatic to bone. Exp. Hematol. Oncol..

[26] Humm J.L., Sartor O., Parker C., Bruland O.S., Macklis R. (2015). Radium-223 in the treatment of osteoblastic metastases: A critical clinical review. Int. J. Radiat. Oncol. Biol. Phys..

[27] Pharmacopoeia E. (2014). European Medicine Agency, London. http://www.ema.europa.eu.

[28] Vaidyanathan G., Zalutsky M.R. (2011). Applications of 211At and 223Ra in targeted alpha-particle radiotherapy. Curr. Radiopharm..

[29] Arazi L., Cooks T., Schmidt M., Keisari Y., Kelson I. (2007). Treatment of solid tumors by interstitial release of recoiling short-lived alpha emitters. Phys. Med. Biol..

[30] Cooks T., Tal M., Raab S., Efrati M., Reitkopf S., Lazarov E., Etzyoni R., Schmidt M., Arazi L., Kelson I. (2012). Intratumoral 224Ra-loaded wires spread alpha-emitters inside solid human tumors in athymic mice achieving tumor control. Anticancer Res..

[31] Li R.G., Napoli E., Jorstad I.S., Bønsdorff T.B., Juzeniene A., Bruland Ø.S., Larsen R.H., Westrøm S. (2021). Calcium Carbonate Microparticles as Carriers of 224Ra: Impact of Specific Activity in Mice with Intraperitoneal Ovarian Cancer. Curr. Radiopharm..

[32] Priest N.D., Dauer L.T., Hoel D.G. (2020). Administration of lower doses of radium-224 to ankylosing spondylitis patients results in no evidence of significant overall detriment. PLoS ONE.

[33] Tiepolt C., Grüning T., Franke W.G. (2002). Renaissance of 224 Ra for the treatment of ankylosing spondylitis: Clinical experiences. Nucl. Med. Commun..

[34] Reitkopf-Brodutch S., Confino H., Schmidt M., Cooks T., Efrati M., Arazi L., Rath-Wolfson L., Marshak G., Kelson I., Keisari Y. (2015). Ablation of experimental colon cancer by intratumoral 224Radium-loaded wires is mediated by alpha particles released from atoms which spread in the tumor and can be augmented by chemotherapy. Int. J. Radiat. Biol..

[35] Li R.G., Stenberg V.Y., Larsen R.H. (2023). An Experimental Generator for Production of High-Purity 212Pb for Use in Radiopharmaceuticals. J. Nucl. Med..

[36] Bé M.-M., Chisté V., Dulieu C., Browne E., Chechev V., Kuzmenko N., Helmer R., Nichols A., Schönfeld E., Dersch R. (2004). Table of Radionuclides (Vol. 2 – A = 151 to 242).

[37] Guérard F., Gestin J.F., Brechbiel M.W. (2013). Production of [(211)At]-astatinated radiopharmaceuticals and applications in targeted α-particle therapy. Cancer Biother. Radiopharm..

[38] Nelson B.J.B., Andersson J.D., Wuest F. (2020). Targeted Alpha Therapy: Progress in Radionuclide Production, Radiochemistry, and Applications. Pharmaceutics.

[39] Lassmann M., Eberlein U. (2022). Comparing absorbed doses and radiation risk of the α-emitting bone-seekers [^223^Ra]RaCl_2_ and [^224^Ra]RaCl_2_. Front. Med..

[40] Hassfjell S.P., Hoff P., Bruland Ø.S., Alstad J. (1994). 212Pb/212Bi-EDTMP—Synthesis and biodistribution of a novel bone seeking alpha-emitting radiopharmaceutical. J. Label. Compd. Radiopharm..

[41] Kang C.S., Song H.A., Milenic D.E., Baidoo K.E., Brechbiel M.W., Chong H.S. (2013). Preclinical evaluation of NETA-based bifunctional ligand for radioimmunotherapy applications using 212Bi and 213Bi: Radiolabeling, serum stability, and biodistribution and tumor uptake studies. Nucl. Med. Biol..

[42] Feng Y., Zalutsky M.R. (2021). Production, purification and availability of 211At: Near term steps towards global access. Nucl. Med. Biol..

[43] Westrøm S., Bønsdorff T.B., Bruland Ø.S., Larsen R.H. (2018). Therapeutic Effect of α-Emitting 224Ra-Labeled Calcium Carbonate Microparticles in Mice with Intraperitoneal Ovarian Cancer. Transl. Oncol..

[44] Jadvar H. (2020). Targeted α-Therapy in Cancer Management: Synopsis of Preclinical and Clinical Studies. Cancer Biother. Radiopharm..

[45] O’Sullivan J.M. (2018). Radium-223 from bench to bedside—Future directions for targeted alpha therapy. Eur. Oncol. Hematol..

[46] Abou D.S., Ulmert D., Doucet M., Hobbs R.F., Riddle R.C., Thorek D.L. (2016). Whole-Body and Microenvironmental Localization of Radium-223 in Naïve and Mouse Models of Prostate Cancer Metastasis. J. Natl. Cancer Inst..

[47] Larsen R.H., Saxtorph H., Skydsgaard M., Borrebaek J., Jonasdottir T.J., Bruland O.S., Klastrup S., Harling R., Ramdahl T. (2006). Radiotoxicity of the alpha-emitting bone-seeker 223Ra injected intravenously into mice: Histology, clinical chemistry and hematology. In Vivo.

[48] Jadvar H., Quinn D.I. (2013). Targeted α-particle therapy of bone metastases in prostate cancer. Clin. Nucl. Med..

[49] Pandit-Taskar N., Larson S.M., Carrasquillo J.A. (2014). Bone-seeking radiopharmaceuticals for treatment of osseous metastases, Part 1: α therapy with 223Ra-dichloride. J. Nucl. Med..

[50] Sgouros G., Roeske J.C., McDevitt M.R., Palm S., Allen B.J., Fisher D.R., Brill A.B., Song H., Howell R.W., Akabani G. (2010). MIRD Pamphlet No. 22 (abridged): Radiobiology and dosimetry of alpha-particle emitters for targeted radionuclide therapy. J. Nucl. Med..

[51] Engle J.W. (2018). The Production of Ac-225. Curr. Radiopharm..

[52] Henriksen G., Fisher D.R., Roeske J.C., Bruland Ø.S., Larsen R.H. (2003). Targeting of osseous sites with alpha-emitting 223Ra: Comparison with the beta-emitter 89Sr in mice. J. Nucl. Med..

[53] Bruland Ø.S., Nilsson S., Fisher D.R., Larsen R.H. (2006). High-linear energy transfer irradiation targeted to skeletal metastases by the alpha-emitter 223Ra: Adjuvant or alternative to conventional modalities. Clin. Cancer Res..

[54] Collins S.M., Pearce A.K., Ferreira K.M., Fenwick A.J., Regan P.H., Keightley J.D. (2015). Direct measurement of the half-life of (223)Ra. Appl. Radiat. Isot..

[55] Shishkin D.N., Krupitskii S.V., Kuznetsov S.A. (2011). Extraction generator of 223Ra for nuclear medicine. Radiochemistry.

[56] Lankoff A., Czerwińska M., Walczak R., Karczmarczyk U., Tomczyk K., Brzóska K., Fracasso G., Garnuszek P., Mikołajczak R., Kruszewski M. (2021). Design and Evaluation of 223Ra-Labeled and Anti-PSMA Targeted NaA Nanozeolites for Prostate Cancer Therapy-Part II. Toxicity, Pharmacokinetics and Biodistribution. Int. J. Mol. Sci..

[57] Chen X., Ji M., Fisher D.R., Wai C.M. (2009). Ionizable Calixarene-Crown Ethers with High Selectivity for Radium over Light Alkaline Earth Metal Ions. Inorg. Chem..

[58] Gott M., Yang P., Kortz U., Stephan H., Pietzsch H.J., Mamat C. (2019). A 224Ra-labeled polyoxopalladate as a putative radiopharmaceutical. Chem. Commun..

[59] Henriksen G., Hoff P., Larsen R.H. (2002). Evaluation of potential chelating agents for radium. Appl. Radiat. Isot..

[60] Kozempel J., Mokhodoeva O., Vlk M. (2018). Progress in Targeted Alpha-Particle Therapy. What We Learned about Recoils Release from In Vivo Generators. Molecules.

[61] Reissig F., Hübner R., Steinbach J., Pietzsch H.J., Mamat C. (2019). Facile preparation of radium-doped, functionalized nanoparticles as carriers for targeted alpha therapy. Inorg. Chem. Front..

[62] Vasiliev A.N., Severin A., Lapshina E., Chernykh E., Ermolaev S., Kalmykov S. (2017). Hydroxyapatite particles as carriers for 223Ra. J. Radioanal. Nucl. Chem..

[63] Abou D.S., Thiele N.A., Gutsche N.T., Villmer A., Zhang H., Woods J.J., Baidoo K.E., Escorcia F.E., Wilson J.J., Thorek D.L.J. (2021). Towards the stable chelation of radium for biomedical applications with an 18-membered macrocyclic ligand. Chem. Sci..

[64] Matyskin A.V., Hansson N.L., Brown P.L., Ekberg C. (2017). Barium and Radium Complexation with Ethylenediaminetetraacetic Acid in Aqueous Alkaline Sodium Chloride Media. J. Solut. Chem..

[65] Pijeira M.S.O., de Menezes A.S., Fechine P.B.A., Shah S.Q., Ilem-Ozdemir D., López E.O., Maricato J.T., Rosa D.S., Ricci-Junior E., Junior S.A. (2022). Folic acid-functionalized graphene quantum dots: Synthesis, characterization, radiolabeling with radium-223 and antiviral effect against Zika virus infection. Eur. J. Pharm. Biopharm..

[66] Fang R.H., Gao W., Zhang L. (2023). Targeting drugs to tumours using cell membrane-coated nanoparticles. Nat. Rev. Clin. Oncol..

[67] Rosenblum D., Joshi N., Tao W., Karp J.M., Peer D. (2018). Progress and challenges towards targeted delivery of cancer therapeutics. Nat. Commun..

[68] Wilhelm S., Tavares A., Dai Q., Ohta S., Audet J., Dvorak H.F., Chan W.C.W. (2016). Analysis of nanoparticle delivery to tumours. Nat. Rev. Mater..

[69] Edis Z., Wang J., Waqas M.K., Ijaz M., Ijaz M. (2021). Nanocarriers-Mediated Drug Delivery Systems for Anticancer Agents: An Overview and Perspectives. Int. J. Nanomed..

[70] Peer D., Karp J.M., Hong S., Farokhzad O.C., Margalit R., Langer R. (2007). Nanocarriers as an emerging platform for cancer therapy. Nat. Nanotechnol..

[71] Vasan N., Baselga J., Hyman D.M. (2019). A view on drug resistance in cancer. Nature.

[72] Davis M.E., Chen Z.G., Shin D.M. (2008). Nanoparticle therapeutics: An emerging treatment modality for cancer. Nat. Rev. Drug Discov..

[73] Mitchell M.J., Billingsley M.M., Haley R.M., Wechsler M.E., Peppas N.A., Langer R. (2021). Engineering precision nanoparticles for drug delivery. Nat. Rev. Drug Discov..

[74] Patra J.K., Das G., Fraceto L.F., Campos E.V.R., Rodriguez-Torres M.D.P., Acosta-Torres L.S., Diaz-Torres L.A., Grillo R., Swamy M.K., Sharma S. (2018). Nano based drug delivery systems: Recent developments and future prospects. J. Nanobiotechnol..

[75] Tsai M., Lu Z., Wang J., Yeh T.K., Wientjes M.G., Au J.L. (2007). Effects of carrier on disposition and antitumor activity of intraperitoneal Paclitaxel. Pharm. Res..

[76] Jian H.J., Wu R.S., Lin T.Y., Li Y.J., Lin H.J., Harroun S.G., Lai J.Y., Huang C.C. (2017). Super-cationic carbon quantum dots synthesized from spermidine as an eye drop formulation for topical treatment of bacterial keratitis. ACS Nano.

[77] Luo L.J., Nguyen D.D., Lai J.Y. (2020). Dually functional hollow ceria nanoparticle platform for intraocular drug delivery: A push beyond the limits of static and dynamic ocular barriers toward glaucoma therapy. Biomaterials.

[78] Hsiao I.L., Huang Y.J. (2011). Effects of various physicochemical characteristics on the toxicities of ZnO and TiO2 nanoparticles toward human lung epithelial cells. Sci. Total Environ..

[79] Nguyen D.D., Lai J.Y. (2022). Synthesis, bioactive properties, and biomedical applications of intrinsically therapeutic nanoparticles for disease treatment. Chem. Eng. J..

[80] Arvizo R.R., Miranda O.R., Moyano D.F., Walden C.A., Giri K., Bhattacharya R., Robertson J.D., Rotello V.M., Reid J.M., Mukherjee P. (2011). Modulating pharmacokinetics, tumor uptake and biodistribution by engineered nanoparticles. PLoS ONE.

[81] Kim S.T., Saha K., Kim C., Rotello V.M. (2013). The role of surface functionality in determining nanoparticle cytotoxicity. Acc. Chem. Res..

[82] Elci S.G., Jiang Y., Yan B., Kim S.T., Saha K., Moyano D.F., Yesilbag T.G., Jackson L.C., Rotello V.M., Vachet R.W. (2016). Surface charge controls the suborgan biodistributions of gold nanoparticles. ACS Nano.

[83] Kostiv U., Patsula V., Šlouf M., Pongrac I.M., Škokić S., Radmilović M.D., Pavičić I., Vrček I.V., Gajović S., Horák D. (2017). Physico-chemical characteristics, biocompatibility, and MRI applicability of novel monodisperse PEG-modified magnetic Fe_3_O_4_ &SiO_2_ core–shell nanoparticles. RSC Adv..

[84] de Oliveira G.M., de Oliveira E.M., Pereira T.C., Papaléo R.M., Bogo M.R. (2017). Implications of exposure to dextran-coated and uncoated iron oxide nanoparticles to developmental toxicity in zebrafish. J. Nanopart. Res..

[85] Bartlett D.W., Davis M.E. (2007). Physicochemical and biological characterization of targeted, nucleic acid-containing nanoparticles. Bioconjug. Chem..

[86] Song E., Zhu P., Lee S.K., Chowdhury D., Kussman S., Dykxhoorn D.M., Feng Y., Palliser D., Weiner D.B., Shankar P. (2005). Antibody mediated in vivo delivery of small interfering RNAs via cell-surface receptors. Nat. Biotechnol..

[87] Su S., M Kang P. (2020). Recent Advances in Nanocarrier-Assisted Therapeutics Delivery Systems. Pharmaceutics.

[88] Blanco E., Shen H., Ferrari M. (2015). Principles of nanoparticle design for overcoming biological barriers to drug delivery. Nat. Biotechnol..

[89] Hong S., Leroueil P.R., Majoros I.J., Orr B.G., Baker J.R., Banaszak Holl M.M. (2007). The binding avidity of a nanoparticle-based multivalent targeted drug delivery platform. Chem. Biol..

[90] Habibi N., Kamaly N., Memic A., Shafiee H. (2016). Self-assembled peptide-based nanostructures: Smart nanomaterials toward targeted drug delivery. Nano Today.

[91] Pawar R., Ben-Ari A., Domb A.J. (2004). Protein and peptide parenteral controlled delivery. Expert Opin. Biol. Ther..

[92] Fan T., Yu X., Shen B., Sun L. (2017). Peptide self-assembled nanostructures for drug delivery applications. J. Nanomater..

[93] Cai L., Wang X., Wang W., Qiu N., Wen J., Duan X., Li X., Chen X., Yang L., Qian Z. (2012). Peptide ligand and PEG-mediated long-circulating liposome targeted to FGFR overexpressing tumor in vivo. Int. J. Nanomed..

[94] Gentili D., Ori G. (2022). Reversible assembly of nanoparticles: Theory, strategies and computational simulations. Nanoscale.

[95] Nie Z., Petukhova A., Kumacheva E. (2010). Properties and emerging applications of self-assembled structures made from inorganic nanoparticles. Nat. Nanotechnol..

[96] Thorkelsson K., Bai P., Xu T. (2015). Self-assembly and applications of anisotropic nanomaterials: A review. Nano Today.

[97] Choi B.D., Cai M., Bigner D.D., Mehta A.I., Kuan C.T., Sampson J.H. (2011). Bispecific antibodies engage T cells for antitumor immunotherapy. Expert Opin. Biol. Ther..

[98] Tang J., Shen D., Zhang J., Ligler F.S., Cheng K. (2015). Bispecific antibodies, nanoparticles and cells: Bringing the right cells to get the job done. Expert Opin. Biol. Ther..

[99] Ye J., Hou B., Chen F., Zhang S., Xiong M., Li T., Xu Y., Xu Z., Yu H. (2022). Bispecific prodrug nanoparticles circumventing multiple immune resistance mechanisms for promoting cancer immunotherapy. Acta Pharm. Sin. B.

[100] Engin A.B., Hayes A.W. (2018). The impact of immunotoxicity in evaluation of the nanomaterials safety. Toxicol. Res..

[101] Lockman P.R., Koziara J.M., Mumper R.J., Allen D.D. (2004). Nanoparticle surface charges alter blood-brain barrier integrity and permeability. J. Drug Target..

[102] Czerwińska M., Fracasso G., Pruszyński M., Bilewicz A., Kruszewski M., Majkowska-Pilip A., Lankoff A. (2020). Design and Evaluation of 223Ra-Labeled and Anti-PSMA Targeted NaA Nanozeolites for Prostate Cancer Therapy-Part I. Materials.

[103] Kihara T., Zhang Y., Hu Y., Mao Q., Tang Y., Miyake J. (2011). Effect of composition, morphology and size of nanozeolite on its in vitro cytotoxicity. J. Biosci. Bioeng..

[104] Męczyńska-Wielgosz S., Piotrowska A., Majkowska-Pilip A., Bilewicz A., Kruszewski M. (2016). Effect of Surface Functionalization on the Cellular Uptake and Toxicity of Nanozeolite A. Nanoscale Res. Lett..

[105] Thomassen L.C., Napierska D., Dinsdale D., Lievens N., Jammaer J., Lison D., Kirschhock C.E., Hoet P.H., Martens J.A. (2012). Investigation of the cytotoxicity of nanozeolites A and Y. Nanotoxicology.

[106] Baken K.A., Vandebriel R.J., Pennings J.L., Kleinjans J.C., van Loveren H. (2007). Toxicogenomics in the assessment of immunotoxicity. Methods.

[107] Fan Y.C., Lee K.D., Tsai Y.C. (2020). Roles of Interleukin-1 Receptor Antagonist in Prostate Cancer Progression. Biomedicines.

[108] Mohammadpour R., Yazdimamaghani M., Cheney D.L., Jedrzkiewicz J., Ghandehari H. (2019). Subchronic toxicity of silica nanoparticles as a function of size and porosity. J. Control Release.

[109] Wang Q., Yang C.S., Ma Z.X., Chen J.C., Zheng J.N., Sun X.Q., Wang J.Q. (2018). Inhibition of prostate cancer DU145 cell growth with small interfering RNA targeting the SATB1 gene. Exp. Ther. Med..

[110] Gardner E.R., Dahut W.L., Scripture C.D., Jones J., Aragon-Ching J.B., Desai N., Hawkins M.J., Sparreboom A., Figg W.D. (2008). Randomized crossover pharmacokinetic study of solvent-based paclitaxel and nab-paclitaxel. Clin. Cancer Res..

[111] Li L., Wartchow C.A., Danthi S.N., Shen Z., Dechene N., Pease J., Choi H.S., Doede T., Chu P., Ning S. (2004). A novel antiangiogenesis therapy using an integrin antagonist or anti-Flk-1 antibody coated 90Y-labeled nanoparticles. Int. J. Radiat. Oncol. Biol. Phys..

[112] Westrøm S., Malenge M., Jorstad I.S., Napoli E., Bruland Ø.S., Bønsdorff T.B., Larsen R.H. (2018). Ra-224 labeling of calcium carbonate microparticles for internal α-therapy: Preparation, stability, and biodistribution in mice. J. Labelled. Comp. Radiopharm..

[113] Kozempel J., Vlk M., Málková E., Bajzíková A., Bárta J., Santos-Oliveira R., Malta Rossi A. (2014). Prospective carriers of 223ra for targeted Alpha Particle therapy. J. Radioanal. Nucl. Chem..

[114] Larsen R.H., Salberg G., Algeta A.S. (2012). Alpha-Emitting Hydroxyapatite Particles. U.S. Patent.

[115] Piotrowska A., Leszczuk E., Bruchertseifer F., Morgenstern A., Bilewicz A. (2013). Functionalized NaA nanozeolites labeled with 224,225 Ra for targeted alpha therapy. J. Nanopart. Res..

[116] Piotrowska A., Męczyńska-Wielgosz S., Majkowska-Pilip A., Koźmiński P., Wójciuk G., Cędrowska E., Bruchertseifer F., Morgenstern A., Kruszewski M., Bilewicz A. (2017). Nanozeolite bioconjugates labeled with 223Ra for targeted alpha therapy. Nucl. Med. Biol..

[117] Reissig F., Bauer D., Ullrich M., Kreller M., Pietzsch J., Mamat C., Kopka K., Pietzsch H.J., Walther M. (2020). Recent Insights in Barium-131 as a Diagnostic Match for Radium-223: Cyclotron Production, Separation, Radiolabeling, and Imaging. Pharmaceuticals.

[118] Rojas J.V., Woodward J.D., Chen N., Rondinone A.J., Castano C.H., Mirzadeh S. (2015). Synthesis and characterization of lanthanum phosphate nanoparticles as carriers for (223)Ra and (225)Ra for targeted alpha therapy. Nucl. Med. Biol..

[119] Rosenberg Y.O., Sade Z., Ganor J. (2018). The precipitation of gypsum, celestine, and barite and coprecipitation of radium during seawater evaporation. Geochim. Cosmochim..

[120] Henriksen G., Schoultz B.W., Michaelsen T.E., Bruland Ø.S., Larsen R.H. (2004). Sterically stabilized liposomes as a carrier for alpha-emitting radium and actinium radionuclides. Nucl. Med. Biol..

[121] Jonasdottir T.J., Fisher D.R., Borrebaek J., Bruland O.S., Larsen R.H. (2006). First In Vivo evaluation of liposome-encapsulated 223 Ra as a potential alpha-particle-emitting cancer therapeutic agent. Anticancer Res..

[122] Pijeira M.S.O., Viltres H., Kozempel J., Sakmár M., Vlk M., İlem-Özdemir D., Ekinci M., Srinivasan S., Rajabzadeh A.R., Ricci-Junior E. (2022). Radiolabeled nanomaterials for biomedical applications: Radiopharmacy in the era of nanotechnology. EJNMMI Radiopharm. Chem..

[123] Kukleva E., Suchánková P., Štamberg K., Vlk M., Šlouf M., Kozempel J. (2019). Surface protolytic property characterization of hydroxyapatite and titanium dioxide nanoparticles. RSC Adv..

[124] Mokhodoeva O., Vlk M., Málková E., Kukleva E., Mičolová P., Štamberg K., Šlouf M., Dzhenloda R., Kozempel J. (2016). Study of 223RA uptake mechanism by fe3o4 nanoparticles: Towards new prospective Theranostic spions. J. Nanopart. Res..

[125] Suchánková P., Kukleva E., Nykl E., Nykl P., Sakmár M., Vlk M., Kozempel J. (2020). Hydroxyapatite and Titanium Dioxide Nanoparticles: Radiolabelling and In Vitro Stability of Prospective Theranostic Nanocarriers for 223Ra and 99mTc. Nanomaterials.

[126] Suchánková P., Kukleva E., Štamberg K., Nykl P., Sakmár M., Vlk M., Kozempel J. (2020). Determination, Modeling and Evaluation of Kinetics of 223Ra Sorption on Hydroxyapatite and Titanium Dioxide Nanoparticles. Materials.

[127] Gawęda W., Pruszyński M., Cędrowska E., Rodak M., Majkowska-Pilip A., Gaweł D., Bruchertseifer F., Morgenstern A., Bilewicz A. (2020). Trastuzumab Modified Barium Ferrite Magnetic Nanoparticles Labeled with Radium-223: A New Potential Radiobioconjugate for Alpha Radioimmunotherapy. Nanomaterials.

[128] Kazakov A.G., Garashchenko B.L., Yakovlev R.Y., Vinokurov S.E., Kalmykov S.N., Myasoedov B.F. (2020). An experimental study of sorption/desorption of selected radionuclides on carbon nanomaterials: A quest for possible applications in future nuclear medicine. Diam. Relat. Mater..

[129] Toro-González M., Dame A.N., Mirzadeh S., Rojas J.V. (2020). Encapsulation and retention of 225Ac, 223Ra, 227Th, and decay daughters in zircon-type gadolinium vanadate nanoparticles. Radiochim. Acta.

[130] Yang Y., Alencar L.M.R., Pijeira M.S.O., Batista B.D.S., França A.R.S., Rates E.R.D., Lima R.C., Gemini-Piperni S., Santos-Oliveira R. (2022). [223Ra] RaCl2 nanomicelles showed potent effect against osteosarcoma: Targeted alpha therapy in the nanotechnology era. Drug Deliv..

[131] Volodkin D.V., Petrov A.I., Prevot M., Sukhorukov G.B. (2004). Matrix polyelectrolyte microcapsules: New system for macromolecule encapsulation. Langmuir.

[132] Napoli E., Bønsdorff T.B., Jorstad I.S., Bruland Ø.S., Larsen R.H., Westrøm S. (2021). Radon-220 diffusion from 224Ra-labeled calcium carbonate microparticles: Some implications for radiotherapeutic use. PLoS ONE.

[133] Alsbeih G., Torres M., Al-Harbi N., Alsubael M. (2004). Loss of wild-type Trp53 protein in mouse fibroblasts leads to increased radioresistance with consequent decrease in repair of potentially lethal damage. Radiat. Res..

[134] Gudkov A.V., Komarova E.A. (2003). The role of p53 in determining sensitivity to radiotherapy. Nat. Rev. Cancer.

[135] Mirzayans R., Andrais B., Scott A., Wang Y.W., Murray D. (2013). Ionizing radiation-induced responses in human cells with differing TP53 status. Int. J. Mol. Sci..

[136] McIlwrath A.J., Vasey P.A., Ross G.M., Brown R. (1994). Cell cycle arrests and radiosensitivity of human tumor cell lines: Dependence on wild-type p53 for radiosensitivity. Cancer Res..

[137] Takagi M., Absalon M.J., McLure K.G., Kastan M.B. (2005). Regulation of p53 translation and induction after DNA damage by ribosomal protein L26 and nucleolin. Cell.

[138] Brooks C.L., Gu W. (2006). p53 ubiquitination: Mdm2 and beyond. Mol. Cell.

[139] Schlereth K., Charles J.P., Bretz A.C., Stiewe T. (2010). Life or death: p53-induced apoptosis requires DNA binding cooperativity. Cell Cycle.

[140] Lee C.L., Blum J.M., Kirsch D.G. (2013). Role of p53 in regulating tissue response to radiation by mechanisms independent of apoptosis. Transl. Cancer Res..

[141] Lowe S.W., Ruley H.E., Jacks T., Housman D.E. (1993). p53-dependent apoptosis modulates the cytotoxicity of anticancer agents. Cell.

[142] Lowe S.W., Bodis S., McClatchey A., Remington L., Ruley H.E., Fisher D.E., Housman D.E., Jacks T. (1994). p53 status and the efficacy of cancer therapy in vivo. Science.

[143] Nikiforov M.A., Hagen K., Ossovskaya V.S., Connor T.M., Lowe S.W., Deichman G.I., Gudkov A.V. (1996). p53 modulation of anchorage independent growth and experimental metastasis. Oncogene.

[144] Larsen R.H., Hoff P., Vergote I.B., Bruland O.S., Aas M., De Vos L., Nustad K. (1995). Alpha-particle radiotherapy with 211At-labeled monodisperse polymer particles, 211At-labeled IgG proteins, and free 211At in a murine intraperitoneal tumor model. Gynecol. Oncol..

[145] Arazi L. (2020). Diffusing alpha-emitters radiation therapy: Approximate modeling of the macroscopic alpha particle dose of a point source. Phys. Med. Biol..

[146] Lazarov E., Arazi L., Efrati M., Cooks T., Schmidt M., Keisari Y., Kelson I. (2012). Comparative in vitro microdosimetric study of murine- and human-derived cancer cells exposed to alpha particles. Radiat. Res..

[147] Popovtzer A., Rosenfeld E., Mizrachi A., Bellia S.R., Ben-Hur R., Feliciani G., Sarnelli A., Arazi L., Deutsch L., Kelson I. (2020). Initial Safety and Tumor Control Results From a "First-in-Human" Multicenter Prospective Trial Evaluating a Novel Alpha-Emitting Radionuclide for the Treatment of Locally Advanced Recurrent Squamous Cell Carcinomas of the Skin and Head and Neck. Int. J. Radiat. Oncol. Biol. Phys..

[148] Keisari Y., Kelson I. (2021). The Potentiation of Anti-Tumor Immunity by Tumor Abolition with Alpha Particles, Protons, or Carbon Ion Radiation and Its Enforcement by Combination with Immunoadjuvants or Inhibitors of Immune Suppressor Cells and Checkpoint Molecules. Cells.

[149] Lloyd R.D., Mays C.W., Taylor G.N., Atherton D.R., Bruenger F.W., Jones C.W. (1982). Radium-224 retention, distribution, and dosimetry in beagles. Radiat. Res..

[150] Milenic D.E., Molinolo A.A., Solivella M.S., Banaga E., Torgue J., Besnainou S., Brechbiel M.W., Baidoo K.E. (2015). Toxicological Studies of 212Pb Intravenously or Intraperitoneally Injected into Mice for a Phase 1 Trial. Pharmaceuticals.

[151] Sgouros G., Hobbs R., Josefsson A. (2018). Dosimetry and Radiobiology of Alpha-Particle Emitting Radionuclides. Curr. Radiopharm..

[152] Decay Data Evaluation Project Atomic and Nuclear Data. http://www.lnhb.fr/nuclear-data/nuclear-data-table/.

[153] Wick R.R., Gössner W. (1993). History and current uses of 224Ra in ankylosing spondylitis and other diseases. Environ. Int..

[154] Arazi L., Cooks T., Schmidt M., Keisari Y., Kelson I. (2010). The treatment of solid tumors by alpha emitters released from (224)Ra-loaded sources-internal dosimetry analysis. Phys. Med. Biol..

[155] Kokov K.V., Egorova B.V., German M.N., Klabukov I.D., Krasheninnikov M.E., Larkin-Kondrov A.A., Makoveeva K.A., Ovchinnikov M.V., Sidorova M.V., Chuvilin D.Y. (2022). 212Pb: Production Approaches and Targeted Therapy Applications. Pharmaceutics.

[156] Seidl C., Zöckler C., Beck R., Quintanilla-Martinez L., Bruchertseifer F., Senekowitsch-Schmidtke R. (2011). 177Lu-immunotherapy of experimental peritoneal carcinomatosis shows comparable effectiveness to 213Bi-immunotherapy, but causes toxicity not observed with 213Bi. Eur. J. Nucl. Med. Mol. Imaging.

[157] Vergote I., Larsen R.H., de Vos L., Nesland J.M., Bruland O., Bjørgum J., Alstad J., Tropé C., Nustad K. (1992). Therapeutic efficacy of the alpha-emitter 211At bound on microspheres compared with 90Y and 32P colloids in a murine intraperitoneal tumor model. Gynecol. Oncol..

[158] Andersson H., Cederkrantz E., Bäck T., Divgi C., Elgqvist J., Himmelman J., Horvath G., Jacobsson L., Jensen H., Lindegren S. (2009). Intraperitoneal alpha-particle radioimmunotherapy of ovarian cancer patients: Pharmacokinetics and dosimetry of (211)At-MX35 F(ab’)2—A phase I study. J. Nucl. Med..

[159] Hallqvist A., Bergmark K., Bäck T., Andersson H., Dahm-Kähler P., Johansson M., Lindegren S., Jensen H., Jacobsson L., Hultborn R. (2019). Intraperitoneal α-Emitting Radioimmunotherapy with 211At in Relapsed Ovarian Cancer: Long-Term Follow-up with Individual Absorbed Dose Estimations. J. Nucl. Med..

[160] Meredith R.F., Torgue J.J., Rozgaja T.A., Banaga E.P., Bunch P.W., Alvarez R.D., Straughn J.M., Dobelbower M.C., Lowy A.M. (2018). Safety and Outcome Measures of First-in-Human Intraperitoneal α Radioimmunotherapy With 212Pb-TCMC-Trastuzumab. Am. J. Clin. Oncol..

[161] Kohane D.S., Tse J.Y., Yeo Y., Padera R., Shubina M., Langer R. (2006). Biodegradable polymeric microspheres and nanospheres for drug delivery in the peritoneum. J. Biomed. Mater Res. A.

[162] Armstrong D.K., Fleming G.F., Markman M., Bailey H.H. (2006). A phase I trial of intraperitoneal sustained-release paclitaxel microspheres (Paclimer) in recurrent ovarian cancer: A Gynecologic Oncology Group study. Gynecol. Oncol..

[163] Boria A.J., Perez-Torres C.J. (2020). Impact of mouse strain and sex when modeling radiation necrosis. Radiat. Oncol..

[164] Iwakawa M., Noda S., Ohta T., Ohira C., Lee R., Goto M., Wakabayashi M., Matsui Y., Harada Y., Imai T. (2003). Different radiation susceptibility among five strains of mice detected by a skin reaction. J. Radiat. Res..

[165] Yang L., Yang J., Li G., Li Y., Wu R., Cheng J., Tang Y. (2017). Pathophysiological Responses in Rat and Mouse Models of Radiation-Induced Brain Injury. Mol. Neurobiol..

[166] Huang Q.T., Zhou L., Zeng W.J., Ma Q.Q., Wang W., Zhong M., Yu Y.H. (2017). Prognostic Significance of Neutrophil-to-Lymphocyte Ratio in Ovarian Cancer: A Systematic Review and Meta-Analysis of Observational Studies. Cell. Physiol. Biochem..

[167] Coosemans A.N., Baert T., D’Heygere V., Wouters R., DE Laet L., VAN Hoylandt A., Thirion G., Ceusters J., Laenen A., Vandecaveye V. (2019). Increased Immunosuppression Is Related to Increased Amounts of Ascites and Inferior Prognosis in Ovarian Cancer. Anticancer Res..

[168] Taylor R.M., Severns V., Brown D.C., Bisoffi M., Sillerud L.O. (2012). Prostate cancer targeting motifs: Expression of αν β3, neurotensin receptor 1, prostate specific membrane antigen, and prostate stem cell antigen in human prostate cancer cell lines and xenografts. Prostate.

[169] Ghosh A., Wang X., Klein E., Heston W.D. (2005). Novel role of prostate-specific membrane antigen in suppressing prostate cancer invasiveness. Cancer Res..

[170] Lütje S., van Rij C.M., Franssen G.M., Fracasso G., Helfrich W., Eek A., Oyen W.J., Colombatti M., Boerman O.C. (2015). Targeting human prostate cancer with 111In-labeled D2B IgG, F(ab’)2 and Fab fragments in nude mice with PSMA-expressing xenografts. Contrast Media Mol. Imaging.

[171] Golombek S.K., May J.N., Theek B., Appold L., Drude N., Kiessling F., Lammers T. (2018). Tumor targeting via EPR: Strategies to enhance patient responses. Adv. Drug Deliv. Rev..

[172] Bolkestein M., de Blois E., Koelewijn S.J., Eggermont A.M., Grosveld F., de Jong M., Koning G.A. (2016). Investigation of Factors Determining the Enhanced Permeability and Retention Effect in Subcutaneous Xenografts. J. Nucl. Med..

[173] Cędrowska E., Pruszyński M., Gawęda W., Żuk M., Krysiński P., Bruchertseifer F., Morgenstern A., Karageorgou M.A., Bouziotis P., Bilewicz A. (2020). Trastuzumab Conjugated Superparamagnetic Iron Oxide Nanoparticles Labeled with 225Ac as a Perspective Tool for Combined α-Radioimmunotherapy and Magnetic Hyperthermia of HER2-Positive Breast Cancer. Molecules.

[174] Chattopadhyay N., Fonge H., Cai Z., Scollard D., Lechtman E., Done S.J., Pignol J.P., Reilly R.M. (2012). Role of antibody-mediated tumor targeting and route of administration in nanoparticle tumor accumulation in vivo. Mol. Pharm..

[175] Dhaliwal A., Zheng G. (2019). Improving accessibility of EPR-insensitive tumor phenotypes using EPR-adaptive strategies: Designing a new perspective in nanomedicine delivery. Theranostics.

[176] Hashizume H., Baluk P., Morikawa S., McLean J.W., Thurston G., Roberge S., Jain R.K., McDonald D.M. (2000). Openings between defective endothelial cells explain tumor vessel leakiness. Am. J. Pathol..

[177] Koukourakis M.I., Koukouraki S., Giatromanolaki A., Archimandritis S.C., Skarlatos J., Beroukas K., Bizakis J.G., Retalis G., Karkavitsas N., Helidonis E.S. (1999). Liposomal doxorubicin and conventionally fractionated radiotherapy in the treatment of locally advanced non-small-cell lung cancer and head and neck cancer. J. Clin. Oncol..

[178] Leporatti S. (2022). Thinking about Enhanced Permeability and Retention Effect (EPR). J. Pers. Med..

[179] Wu J. (2021). The Enhanced Permeability and Retention (EPR) Effect: The Significance of the Concept and Methods to Enhance Its Application. J. Pers. Med..

[180] Maeda H. (2013). The link between infection and cancer: Tumor vasculature, free radicals, and drug delivery to tumors via the EPR effect. Cancer Sci..

[181] Maeda H., Nakamura H., Fang J. (2013). The EPR effect for macromolecular drug delivery to solid tumors: Improvement of tumor uptake, lowering of systemic toxicity, and distinct tumor imaging in vivo. Adv. Drug Deliv. Rev..

[182] Maeda H. (2010). Nitroglycerin enhances vascular blood flow and drug delivery in hypoxic tumor tissues: Analogy between angina pectoris and solid tumors and enhancement of the EPR effect. J. Control. Release.

[183] Sano K., Nakajima T., Choyke P.L., Kobayashi H. (2012). Markedly enhanced permeability and retention effects induced by photo-immunotherapy of tumors. ACS Nano.

[184] Bertrand N., Grenier P., Mahmoudi M., Lima E.M., Appel E.A., Dormont F., Lim J.M., Karnik R., Langer R., Farokhzad O.C. (2017). Mechanistic understanding of in vivo protein corona formation on polymeric nanoparticles and impact on pharmacokinetics. Nat. Commun..

[185] Chinen A.B., Guan C.M., Ko C.H., Mirkin C.A. (2017). The Impact of Protein Corona Formation on the Macrophage Cellular Uptake and Biodistribution of Spherical Nucleic Acids. Small.

[186] Reddy L.H., Arias J.L., Nicolas J., Couvreur P. (2012). Magnetic nanoparticles: Design and characterization, toxicity and biocompatibility, pharmaceutical and biomedical applications. Chem. Rev..

[187] Baldi G., Ravagli C., Mazzantini F., Loudos G., Adan J., Masa M., Psimadas D., Fragogeorgi E.A., Locatelli E., Innocenti C. (2014). In vivo anticancer evaluation of the hyperthermic efficacy of anti-human epidermal growth factor receptor-targeted PEG-based nanocarrier containing magnetic nanoparticles. Int. J. Nanomed..

[188] Turner P.G., O’Sullivan J.M. (2015). (223)Ra and other bone-targeting radiopharmaceuticals-the translation of radiation biology into clinical practice. Br. J. Radiol..

[189] Hofman M.S., Violet J., Hicks R.J., Ferdinandus J., Thang S.P., Akhurst T., Iravani A., Kong G., Ravi Kumar A., Murphy D.G. (2018). [177Lu]-PSMA-617 radionuclide treatment in patients with metastatic castration-resistant prostate cancer (LuPSMA trial): A single-centre, single-arm, phase 2 study. Lancet Oncol..

[190] Ahmadzadehfar H., Essler M., Schäfers M., Rahbar K. (2016). Radioligand therapy with 177Lu-PSMA-617 of metastatic prostate cancer has already been arrived in clinical use. Nucl. Med. Biol..

[191] Dorff T.B., Fanti S., Farolfi A., Reiter R.E., Sadun T.Y., Sartor O. (2019). The Evolving Role of Prostate-Specific Membrane Antigen-Based Diagnostics and Therapeutics in Prostate Cancer. Am. Soc. Clin. Oncol. Educ. Book.

[192] Emmett L., Willowson K., Violet J., Shin J., Blanksby A., Lee J. (2017). Lutetium 177 PSMA radionuclide therapy for men with prostate cancer: A review of the current literature and discussion of practical aspects of therapy. J. Med. Radiat. Sci..

[193] Kratochwil C., Giesel F.L., Stefanova M., Benešová M., Bronzel M., Afshar-Oromieh A., Mier W., Eder M., Kopka K., Haberkorn U. (2016). PSMA-Targeted Radionuclide Therapy of Metastatic Castration-Resistant Prostate Cancer with 177Lu-Labeled PSMA-617. J. Nucl. Med..

[194] Sun M., Zhang J., Liang S., Du Z., Liu J., Sun Z., Duan J. (2021). Metabolomic characteristics of hepatotoxicity in rats induced by silica nanoparticles. Ecotoxicol. Environ. Saf..

[195] Brito A.E., Etchebehere E. (2020). Radium-223 as an Approved Modality for Treatment of Bone Metastases. Semin. Nucl. Med..

[196] Satapathy S., Sood A., Das C.K., Mittal B.R. (2021). Evolving role of 225Ac-PSMA radioligand therapy in metastatic castration-resistant prostate cancer-a systematic review and meta-analysis. Prostate Cancer Prostatic Dis..

[197] Sathekge M., Knoesen O., Meckel M., Modiselle M., Vorster M., Marx S. (2017). 213Bi-PSMA-617 targeted alpha-radionuclide therapy in metastatic castration-resistant prostate cancer. Eur. J. Nucl. Med. Mol. Imaging.

[198] Gabizon A., Shmeeda H., Barenholz Y. (2003). Pharmacokinetics of pegylated liposomal Doxorubicin: Review of animal and human studies. Clin. Pharmacokinet..

[199] Nilsson S., Larsen R.H., Fosså S.D., Balteskard L., Borch K.W., Westlin J.E., Salberg G., Bruland O.S. (2005). First clinical experience with alpha-emitting radium-223 in the treatment of skeletal metastases. Clin. Cancer Res..

[200] Gabizon A., Tzemach D., Mak L., Bronstein M., Horowitz A.T. (2002). Dose dependency of pharmacokinetics and therapeutic efficacy of pegylated liposomal doxorubicin (DOXIL) in murine models. J. Drug Target..

[201] Gabizon A.A. (2001). Stealth liposomes and tumor targeting: One step further in the quest for the magic bullet. Clin. Cancer Res..

[202] Tan C., Wang J., Sun B. (2021). Biopolymer-liposome hybrid systems for controlled delivery of bioactive compounds: Recent advances. Biotechnol. Adv..

[203] Sarabandi K., Jafari S.M. (2020). Effect of chitosan coating on the properties of nanoliposomes loaded with flaxseed-peptide fractions: Stability during spray-drying. Food Chem..

[204] Ternullo S., Werning L.V.S., Holsæter A.M., Škalko-Basnet N. (2019). Curcumin-in-deformable liposomes-in-chitosan-hydrogel as a novel wound dressing. Pharmaceutics.

[205] Yu J.Y., Chuesiang P., Shin G.H., Park H.J. (2021). Post-Processing Techniques for the Improvement of Liposome Stability. Pharmaceutics.

[206] van den Hoven J.M., Metselaar J.M., Storm G., Beijnen J.H., Nuijen B. (2012). Cyclodextrin as membrane protectant in spray-drying and freeze-drying of PEGylated liposomes. Int. J. Pharm..

[207] Bloomer W.D., McLaughlin W.H., Neirinckx R.D., Adelstein S.J., Gordon P.R., Ruth T.J., Wolf A.P. (1981). Astatine-211—Tellurium radiocolloid cures experimental malignant ascites. Science.

[208] Rotmensch J., Atcher R.W., Schlenker R., Hines J., Grdina D., Block B.S., Press M.F., Herbst A.L., Weichselbaum R.R. (1989). The effect of the alpha-emitting radionuclide lead-212 on human ovarian carcinoma: A potential new form of therapy. Gynecol. Oncol..

[209] Borchardt P.E., Yuan R.R., Miederer M., McDevitt M.R., Scheinberg D.A. (2003). Targeted actinium-225 in vivo generators for therapy of ovarian cancer. Cancer Res..

[210] Heyerdahl H., Abbas N., Sponheim K., Mollatt C., Bruland Ø., Dahle J. (2013). Targeted alpha therapy with 227Th-trastuzumab of intraperitoneal ovarian cancer in nude mice. Curr. Radiopharm..

[211] Milenic D.E., Baidoo K.E., Kim Y.S., Barkley R., Brechbiel M.W. (2017). Comparative studies on the therapeutic benefit of targeted α-particle radiation therapy for the treatment of disseminated intraperitoneal disease. Dalton Trans..

[212] Murray I., Rojas B., Gear J., Callister R., Cleton A., Flux G.D. (2020). Quantitative Dual-Isotope Planar Imaging of Thorium-227 and Radium-223 Using Defined Energy Windows. Cancer Biother. Radiopharm..

[213] Pommé S., Marouli M., Suliman G., Dikmen H., Van Ammel R., Jobbágy V., Dirican A., Stroh H., Paepen J., Bruchertseifer F. (2012). Measurement of the 225Ac half-life. Appl. Radiat. Isot..

[214] Derrien A., Gouard S., Maurel C., Gaugler M.H., Bruchertseifer F., Morgenstern A., Faivre-Chauvet A., Classe J.M., Chérel M. (2015). Therapeutic Efficacy of Alpha-RIT Using a (213)Bi-Anti-hCD138 Antibody in a Mouse Model of Ovarian Peritoneal Carcinomatosis. Front. Med..

[215] Milenic D.E., Baidoo K.E., Kim Y.S., Barkley R., Brechbiel M.W. (2017). Targeted α-Particle Radiation Therapy of HER1-Positive Disseminated Intraperitoneal Disease: An Investigation of the Human Anti-EGFR Monoclonal Antibody, Panitumumab. Transl. Oncol..

[216] Boudousq V., Bobyk L., Busson M., Garambois V., Jarlier M., Charalambatou P., Pèlegrin A., Paillas S., Chouin N., Quenet F. (2013). Comparison between internalizing anti-HER2 mAbs and non-internalizing anti-CEA mAbs in alpha-radioimmunotherapy of small volume peritoneal carcinomatosis using 212Pb. PLoS ONE.

[217] Elgqvist J., Andersson H., Bäck T., Hultborn R., Jensen H., Karlsson B., Lindegren S., Palm S., Warnhammar E., Jacobsson L. (2005). Therapeutic efficacy and tumor dose estimations in radioimmunotherapy of intraperitoneally growing OVCAR-3 cells in nude mice with (211)At-labeled monoclonal antibody MX35. J. Nucl. Med..

[218] Kasten B.B., Arend R.C., Katre A.A., Kim H., Fan J., Ferrone S., Zinn K.R., Buchsbaum D.J. (2017). B7-H3-targeted 212Pb radioimmunotherapy of ovarian cancer in preclinical models. Nucl. Med. Biol..

[219] Milenic D., Garmestani K., Dadachova E., Chappell L., Albert P., Hill D., Schlom J., Brechbiel M. (2004). Radioimmunotherapy of human colon carcinoma xenografts using a 213Bi-labeled domain-deleted humanized monoclonal antibody. Cancer Biother. Radiopharm..

[220] Milenic D.E., Garmestani K., Brady E.D., Albert P.S., Ma D., Abdulla A., Brechbiel M.W. (2005). Alpha-particle radioimmunotherapy of disseminated peritoneal disease using a (212)Pb-labeled radioimmunoconjugate targeting HER2. Cancer Biother. Radiopharm..

[221] Palm S., Bäck T., Claesson I., Danielsson A., Elgqvist J., Frost S., Hultborn R., Jensen H., Lindegren S., Jacobsson L. (2007). Therapeutic efficacy of astatine-211-labeled trastuzumab on radioresistant SKOV-3 tumors in nude mice. Int. J. Radiat. Oncol. Biol. Phys..

[222] Ahenkorah S., Cassells I., Deroose C.M., Cardinaels T., Burgoyne A.R., Bormans G., Ooms M., Cleeren F. (2021). Bismuth-213 for Targeted Radionuclide Therapy: From Atom to Bedside. Pharmaceutics.

[223] Asadian S., Mirzaei H., Kalantari B.A., Davarpanah M.R., Mohamadi M., Shpichka A., Nasehi L., Es H.A., Timashev P., Najimi M. (2020). β-radiating radionuclides in cancer treatment, novel insight into promising approach. Pharmacol. Res..

[224] Sofou S. (2008). Radionuclide carriers for targeting of cancer. Int. J. Nanomed..

[225] Kaplan W.D., Zimmerman R.E., Bloomer W.D., Knapp R.C., Adelstein S.J. (1981). Therapeutic intraperitoneal 32P: A clinical assessment of the dynamics of distribution. Radiology.

[226] Vergote I.B., Vergote-De Vos L.N., Abeler V.M., Aas M., Lindegaard M.W., Kjørstad K.E., Tropé C.G. (1992). Randomized trial comparing cisplatin with radioactive phosphorus or whole-abdomen irradiation as adjuvant treatment of ovarian cancer. Cancer.

[227] Mulford D.A., Scheinberg D.A., Jurcic J.G. (2005). The promise of targeted {alpha}-particle therapy. J. Nucl. Med..

[228] Verheijen R.H., Massuger L.F., Benigno B.B., Epenetos A.A., Lopes A., Soper J.T., Markowska J., Vyzula R., Jobling T., Stamp G. (2006). Phase III trial of intraperitoneal therapy with yttrium-90-labeled HMFG1 murine monoclonal antibody in patients with epithelial ovarian cancer after a surgically defined complete remission. J. Clin. Oncol..

[229] Srivastava S.C., Mausner L.F., Baum R. (2014). Therapeutic Radionuclides: Production, Physical Characteristics, and Applications. Therapeutic Nuclear Medicine.

[230] Au J.L., Lu Z., Wientjes M.G. (2015). Versatility of Particulate Carriers: Development of Pharmacodynamically Optimized Drug-Loaded Microparticles for Treatment of Peritoneal Cancer. AAPS J..

[231] Khong A., Cleaver A.L., Fahmi Alatas M., Wylie B.C., Connor T., Fisher S.A., Broomfield S., Lesterhuis W.J., Currie A.J., Lake R.A. (2014). The efficacy of tumor debulking surgery is improved by adjuvant immunotherapy using imiquimod and anti-CD40. BMC Cancer.

[232] Zalutsky M.R., Pruszynski M. (2011). Astatine-211: Production and availability. Curr. Radiopharm..

[233] Morgenstern A., Bruchertseifer F., Apostolidis C. (2012). Bismuth-213 and actinium-225-generator performance and evolving therapeutic applications of two generator-derived alpha-emitting radioisotopes. Curr. Radiopharm..

